# A circadian repressor promotes flowering via dual repression: *PtTOC* suppresses the floral inhibitor *PtTFL2* in *Pinus tabuliformis*

**DOI:** 10.1186/s12870-026-08511-z

**Published:** 2026-03-19

**Authors:** Kai Qu, Huili Wang, Boning Yang, Shihui Niu, Hua Xue, Yousry A. El-Kassaby, Wei Li

**Affiliations:** 1 State Key Laboratory of Tree Genetics and Breeding, National Engineering Research Center of Tree Breeding and Ecological Restoration, College of Biological Sciences and Technology, Beijing Forestry University, Beijing, 100083 China; 2 Institute of Forestry and Pomology, Beijing Academy of Agriculture and Forestry Sciences, Beijing, 100093 China; 3 Department of Forest and Conservation Sciences, Faculty of Forestry, University of British Columbia, Vancouver, British Columbia V6T 1Z4 Canada; 4 College of Forestry, Southwest Forestry University, Kunming, 650224 China

**Keywords:** *Pinus tabuliformis*, Response regulator gene family, TIMING OF CAB EXPRESSION 1, Promoting flowering, Transcriptional repressor

## Abstract

The Response Regulator (RR) gene family functions as a molecular “switch” in plant signal transduction, with *TOC1* (*PRR1*) frequently acting as a transcriptional repressor that integrates both internal and external signals. While RR homologs in gymnosperms differ from those in angiosperms, research on their function remains sparse. This study presents a genome-wide analysis of the RR gene family in *Pinus tabuliformis*, identifying 35 RR genes that are grouped into three subfamilies: A-type ARRs (17 genes), B-type ARRs (16 genes), and pseudo-RRs (2 genes). Except for *PtPRR43*, these genes are distributed across various chromosomes, with 16 displaying broad expression in multiple organs. Our findings revealed that *PtTOC* indirectly promotes flowering by suppressing *PtTFL2*, a floral repressor, suggesting its role in balancing circadian rhythms and environmental signals. Additionally, *PtTOC* overexpression reduces cold tolerance, likely through the downregulation of cold-responsive pathways, suggesting a trade-off between promoting flowering and stress resilience. Subcellular localization analysis suggests *PtTOC* undergoes phosphorylation-regulated phase transitions, highlighting its dynamic role in cellular signaling and adaptation. Collectively, these results position *PtTOC* as a central integrator of environmental signals and circadian regulation, offering insights into strategies for enhancing reproductive performance and resilience in gymnosperms.

**Supplementary Information:**

The online version contains supplementary material available at 10.1186/s12870-026-08511-z.

## Introduction

The Response Regulator (RR) gene family lies at the core of plant survival and adaptation, orchestrating vital processes such as stress responses, circadian rhythms, and flowering time [1, 2]. Acting as key mediators in the two-component signaling system, RR genes translate external stimuli into intracellular responses that regulate downstream gene expression. Through histidine phosphorylation-mediated pathways involving histidine kinases (HK), histidine phosphotransfer proteins (HP), and RR proteins, this signaling cascade enables plants to adapt to environmental fluctuations while maintaining growth homeostasis [1].

The functional diversity and central role of the RR gene family in plant signaling networks make it a major focus of molecular and evolutionary research. Based on structure and conserved domains, RRs are typically classified into three subfamilies: A-Type ARRs (A-ARRs), B-Type ARRs (B-ARRs), and Pseudo-RRs [3]. Each subfamily fulfills distinct roles that contribute to plant adaptation to dynamic internal and external conditions. The A-ARR subfamily, including cytokinin-inducible genes such as *ARR3* and *ARR5*, functions primarily as negative feedback regulators in cytokinin signaling. These genes possess a conserved N-terminal receiver domain and a C-terminal phospho-acceptor site and often act antagonistically with abscisic acid (ABA) pathway, influencing abiotic stress responses [4]. In *Arabidopsis thaliana*, certain A-ARRs negatively regulate adventitious bud induction by inhibiting meristem-promoting genes [5]. In contrast, B-ARRs promote cytokinin-mediated gene expression, playing critical roles in shoot regeneration and immune responses. These proteins contain a Myb-like DNA-binding GARP domain that facilitates transcriptional activation [1, 6].

Compared to A-ARRs and B-ARRs, Pseudo-RRs have historically received less attention, but recent studies have revealed their diverse and essential functions. The five well-characterized pseudo-RRs—*PRR9*, *PRR7*, *PRR5*, *PRR3*, and *TIMING OF CAB EXPRESSION1* (*TOC1*)—are sequentially expressed from dawn to midnight, each playing a distinct role in maintaining circadian rhythms and regulating developmental transitions [7, 8]. These regulators contribute to abiotic stress responses and floral organ development during the transition from vegetative to reproductive growth [9–11]. For example, the “Green Revolution” gene *TaPRR37* (*TaPpd1*) enhances wheat yield by regulating flowering time and plant height, while *TOC1* improves agronomic traits in crops such as wheat and rice by controlling heading date and enhancing pathogen resistance [12]. *TOC1* functions as a universal transcriptional repressor via its CCT domain, maintaining circadian oscillations by inhibiting key transcription factors such as *CIRCADIAN CLOCK-ASSOCIATED 1* (*CCA1*) and *LATE ELONGATED HYPOCOTYL* (*LHY*) [8, 13]. Moreover, *TOC1* integrates environmental cues, such as light and temperature, to fine-tune flowering and improve resilience under variable climatic conditions [13, 14].

Flowering time is tightly regulated through a complex interplay between environmental signals, activators, and repressors [15]. In angiosperms, timely floral initiation depends on the transcriptional activation of *FLOWERING LOCUS T* (*FT*) by *CONSTANS* (*CO*), a process finely tuned by the circadian clock and photoperiodic cues [16]. *TOC1* plays a pivotal role in this regulation, with its nighttime stability controlled by the E3 ubiquitin ligases *ZEITLUPE* (*ZTL*) and *GIGANTEA* (*GI*). *ZTL* forms a complex with *FLAVIN-BINDING KELCH REPEAT F-BOX 1* (*FKF1*) to target *CO*, which in turn promotes the degradation of *TOC1* and *PRR5*, thereby modulating flowering time. In the morning, *CCA1* activates *CYCLING DOF FACTORS* (*CDFs)* that repress *CO* expression, delaying flowering initiation. Pseudo-RRs, such as *TOC1* influence this network by regulating *CDF* expression either directly or indirectly [17–19]. This regulatory framework also involves *TERMINAL FLOWER 1-like* (*TFL1-like*) genes, which belong to the Phosphatidyl Ethanolamine Binding Protein (PEBP) family, alongside *FT*. Although *FT* and *TFL1-like* share high sequence similarity, their functions are antagonistic: *FT* promotes flowering, while *TFL1*-*like* represses it [20]. In *Picea abies*, only two *FT/TFL1-like* genes (*PaFTL1* and *PaFTL2*) have been identified, both acting as flowering repressors when expressed in *A. thaliana* [21]. However, due to limited genomic resources, the regulatory mechanisms governing *TFL1-like* genes in gymnosperms remain largely unknown, though they are likely influenced by core circadian oscillator components. Despite these advances, the precise mechanisms through which *TOC1* integrates environmental signals to regulate flowering in gymnosperms remain poorly understood.

In angiosperms, the RR gene family has expanded and diversified, largely due to whole-genome duplication (WGD) events [22]. In contrast, gymnosperms, which generally lack recent polyploidization, exhibit limited gene family expansion. Whether gymnosperms retain unique, lineage-specific RR subtypes remains unclear. Most RR studies have focused on angiosperms such as *A. thaliana*, *Oryza sativa* (rice), and *Populus trichocarpa* (poplar), while gymnosperms, particularly conifers, have received comparatively little attention [1, 23–26]. Interestingly, in conifers, cytokinins (CKs) can induce adventitious bud formation directly from cotyledons without a dedifferentiation phase, a process accompanied by strong upregulation of RR genes [27]. This highlights the potential of RR studies in conifers to uncover unique regulatory mechanisms and broaden genomic models for gymnosperms. Gymnosperms exhibit distinctive life cycles and exceptional adaptability to diverse and often harsh environments, emphasizing the importance of understanding their molecular regulatory systems. Among them, *Pinus tabuliformis*, a conifer endemic to China, is of major ecological and economic importance, especially across northern regions [28]. Advances in genomic resources have positioned *P. tabuliformis* as a representative model for conifer molecular biology [29]. Its extended growth cycle, unique flowering characteristics, and broad ecological distribution make it an ideal species for studying RR gene evolution and function. Understanding its flowering regulation mechanisms is particularly critical under current climate change pressures, as pseudo-RR genes may mediate its diverse response to environmental cues.

In this study, we performed a comprehensive genome-wide analysis of the RR gene family in *P. tabuliformis*, including classification, expression profiling, and functional prediction. Using an integrative approach combing heterologous expression systems in *A. thaliana*, yeast one-hybrid assays, and in vivo transient expression in *Nicotiana benthamiana*, we elucidated the molecular mechanism by which *PtTOC* acts as transcriptional repressor to regulate flowering time by inhibiting the expression of *PtTFL2*. Furthermore, our results reveal the significant influence of temperature cues on circadian-regulated reproductive development, providing novel insights into the interplay between environmental signaling and genetic regulation in *P. tabuliformis*. These findings deepen our understanding of the molecular mechanisms controlling flowering time in this ecologically and economically important conifer and offer a foundation for future breeding strategies aimed at enhancing resilience and adaptability under changing climatic conditions.

## Materials and methods

### Identification of RR genes in *P. tabuliformis*

Genomic data for *P. tabuliformis* was sourced from the National Gene Bank Life Big Data Platform (CNSA, CNP0001649, https://db.cngb.org/cnsa/), with corresponding genome annotations accessed via Figshare (DOI: 10.6084/m9.figshare.16847146). For comparative analysis, protein sequences of the *A. thaliana* RR family were obtained from the TAIR database (www.arabidopsis.org) (Table S1). This reference set included 10 A-type ARRs (*ARR3*, *4*, *5*, *6*, *7*, *8*, *9*, *15*, *16*, *17*), 11 B-type ARRs (*ARR1*, *2*, *10*, *11*, *12*, *13*, *14*, *18*, *19*, *20*, *21*), and 5 Pseudo-RRs (*TOC1*, *PRR3*, *5*, *7*, *9*) [1].

Putative RR genes in *P. tabuliformis* were identified by aligning the *A. thaliana* sequences against the *P. tabuliformis* proteome using the BLASTP plugin in TBtools [30]. To ensure classification accuracy, conserved domains were verified using the NCBI Conserved Domain Database (CDD) (https://www.ncbi.nlm.nih.gov/Structure/bwrpsb/bwrpsb.cgi) and the Pfam database (http://pfam.janelia.org/). Subfamily assignment was determined by a rigorous hierarchical protocol: (1) primary classification was based on domain architecture (e.g., the presence of a Myb-like DNA-binding GARP domain for Type-B ARRs); (2) phylogenetic clustering with *A. thaliana* homologs provided secondary support; and (3) in instances of conflict between phylogenetic positioning and domain composition, the latter took precedence. This domain-centric classification approach aligns with established standards for plant response regulator research.

### Sequence alignment, physicochemical properties, localization, and structure visualization

Multiple sequence alignments for the RR gene family were performed using the R package ggmsa (v1.3.4). The ExPASy ProtParam tool (https://web.expasy.org/protparam/) provided physicochemical property predictions for the *P. tabuliformis’* RR proteins, and subcellular localization was assessed via the PSORT online tool (https://www.genscript.com/psort.html). Three-dimensional protein structures were accessed from the AlphaFold Protein Structure Database and visualized using PyMOL software (https://pymol.affinitycn.cn) [31].

### Phylogenetic analysis and genomic mapping

Phylogenetic trees were constructed using the neighbor-joining method in MEGA X, with CIRCLE TREE software enhancing visualization (https://www.chiplot.online/circleTree.html). RR protein sequences were sourced from UniProt (www.uniprot.org). Chromosomal localization was determined using MapGene2Chro, and TBtools was employed to analyze gene structures, conserved domains, and promoter cis-regulatory elements. MCScanX software enabled synteny analysis of RR genes among *P. tabuliformis*, *P. trichocarpa*, *A. thaliana*, *Ginkgo biloba* and *Sequoiadendron giganteum*.

### RNA extraction and expression analysis of PtRR genes

Total RNA was extracted from various *P. tabuliformis* samples using Trizol reagent (Invitrogen). Extracted RNA was reverse-transcribed to create cDNA libraries, sequenced on the Illumina NovaSeq platform (paired-end, 2 × 150 bp). Clean reads were aligned to the *P. tabuliformis* reference transcriptome [29]. Expression data covering different tissues, seasonal cycles, diurnal cycles, and photoperiod treatments are detailed in Table S2 and S3. Transcriptome data for shoot apices and needles under various light and temperature conditions are available on NCBI.

### Vector construction and *A. thaliana* transformation

Transgenic *A. thaliana* lines overexpressing *PtTOC* were generated by cloning the gene into the PBI121-eGFP vector under the 35 S promoter. Recombinant plasmids were introduced into *Agrobacterium tumefaciens* strain GV3101 and transformed into *A. thaliana* (*Col-0*) using the floral dip method. Homozygous lines were selected on Murashige and Skoog (MS) medium with kanamycin, with three lines chosen per construct. Due to the lack of a transformation system in *P. tabuliformis*, *A. thaliana* was used as a model for heterologous expression [32]. A full list of primers used is provided in Table S4.

### Subcellular localization

The *PtTOC g*ene was fused with GFP and mCherry. The recombinant plasmid pGD-GFP-*PtTOC* was introduced into *Agrobacterium* strain GV3101 and infiltrated into *N. benthamiana* leaves [33]. Fluorescence was detected using a Leica TCS SP8 confocal microscope three days post-infiltration.

### Yeast one*-*hybrid and dual*-*luciferase assays

For yeast one-hybrid (Y1H) assays, recombinant plasmids were co-transformed into the EGY48 yeast strain. Transformants were cultured at 28 °C on SD/-Trp/-Ura medium for four days, followed by transfer to SD/-Trp/-Ura + Gal + Raf + X-gal medium for an additional three days. Negative controls included various empty vectors [32]. For dual-luciferase assays, the coding sequence of *PtTOC* was cloned into the pGreenII 62-SK vector, while the *PtTFL2* promoter was inserted into the pGreenII0800-LUC vector. These plasmids were co-infiltrated into *N. benthamiana* leaves, and luciferase activity was measured post-incubation [34].

### Histochemical staining

DAB (3,3′-Diaminobenzidine) and NBT (Nitroblue Tetrazolium) staining were conducted on leaves from transgenic and wild-type *A. thaliana* plants. Samples were treated at 4 °C for two weeks, stained for eight hours, washed with PBS (phosphate-buffer saline), and decolorized in 95% ethanol before imaging [35].

### *PtTOC* yeast stress resistance assay

*PtTOC* was cloned into the pYES2-NTB vector and cultured on SD-Ura medium with 2% glucose at 28 °C for three days, verified by colony PCR. Positive clones were induced in SG-Ura medium for 36 h, followed by exposure to varied temperatures for 72 h. Cultures were serially diluted and plated on SG-Ura solid medium for growth observation [36].

## Results

### Identification, characterization, and phylogenetic analysis of RR family in *P. tabuliformis*

In this study, we identified 35 non-redundant members of the RR gene family in *P. tabuliformis* using *A. thaliana* RR gene sequences as reference. Based on a comprehensive analysis of RR domain architectures, these genes were classified into 17 A-type ARR and 16 B-type ARR members. This distribution notably exceeds those reported in angiosperms such as *A. thaliana*, *Zea mays*, and *P. trichocarpa*. Conversely, the Pseudo-RR subfamily comprised only 2 genes, representing a markedly reduced count compared to other plant taxa. The classification of the *PtRR* family prioritized conserved domain composition over phylogenetic positioning. Specifically, genes were assigned to the A-ARR subfamily if they possessed a REC domain lacking a GARP DNA-binding domain; to the B-ARR subfamily if both REC and GARP domains were present; and to the Pseudo-RR subfamily if they harbored a degenerate REC domain (lacking the canonical Asp phosphorylation site) alongside a CCT domain. In instances where phylogenetic clustering conflicted with domain composition—as observed for *PtPRR47*, *PtPRR48*, *PtPRR49*, and *PtPRR50*—domain architecture was utilized as the definitive criterion, ensuring consistency with established genomic classification standards.Gene nomenclature was standardized according to the *P. tabuliformis* genome annotation files (Table S5, S6). The predicted RR proteins varied in molecular weight from 11.33 to 103.12 kDa, with lengths ranging from 100 (*PtPRR13*) to 951 amino acids (*PtPRR4*) (Table S5). In general, A-ARR proteins were shorter than their B-ARR and Pseudo-RR counterparts. Proteins such as *PtPRR43* and *PtTOC* exhibited high theoretical isoelectric points (pI), suggesting functional activity in neutral to mildly alkaline environments. In contrast, *PtPRR6* and *PtPRR9*, had acidic pIs, indicating potential activity in acidic cellular compartments or extracellular environments. Subcellular localization predictions revealed that RR proteins are broadly distributed, primarily in the nucleus and cytoplasm. All RR proteins exhibited negative GRAVY (Grand Average Hydropathicity values) values, classifying them as hydrophilic, while their high aliphatic index suggests structural stability under a range of temperatures, a likely adaptive advantage for conifers inhabiting diverse climatic conditions.

**Fig. 1:**
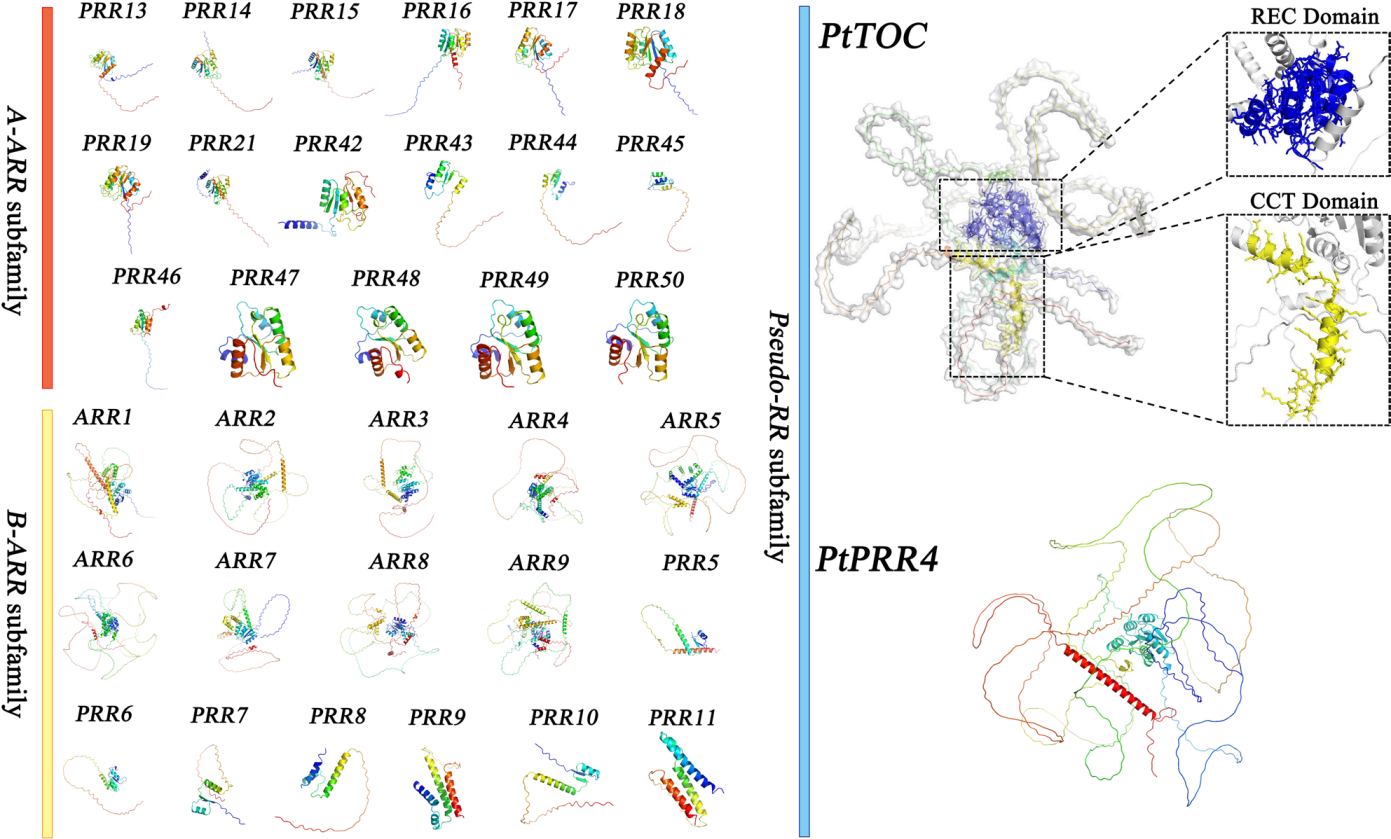
Identification of the RR gene family members in P. tabuliformis. (colored blocks represent genes belonging to distinct subfamilies: red, yellow, and blue indicates A-ARR, B-ARR, and pseudo-RR subfamilies, respectively)

Utilizing AlphaFold3, we predicted the three-dimensional structures of all 35 RR proteins, which were subsequently visualized using PyMOL (Fig. 1). Distinct structural variations among the three subfamilies suggested functional specialization: A-ARR proteins generally exhibited compact, highly folded domains with a pronounced disordered N-terminus region, consistent with their regulatory roles. In contrast, B-ARR proteins contained more disordered regions and fewer canonical domains, reflecting their function as transcription factors in complex signaling networks. The Pseudo-RR proteins displayed intricate structural folds that may enhance their functional interactions. Notably, structural analysis of *PtTOC* revealed the presence of both REC and CCT domains, which are likely essential for mediating protein-protein and protein-DNA interactions.

**Fig. 2:**
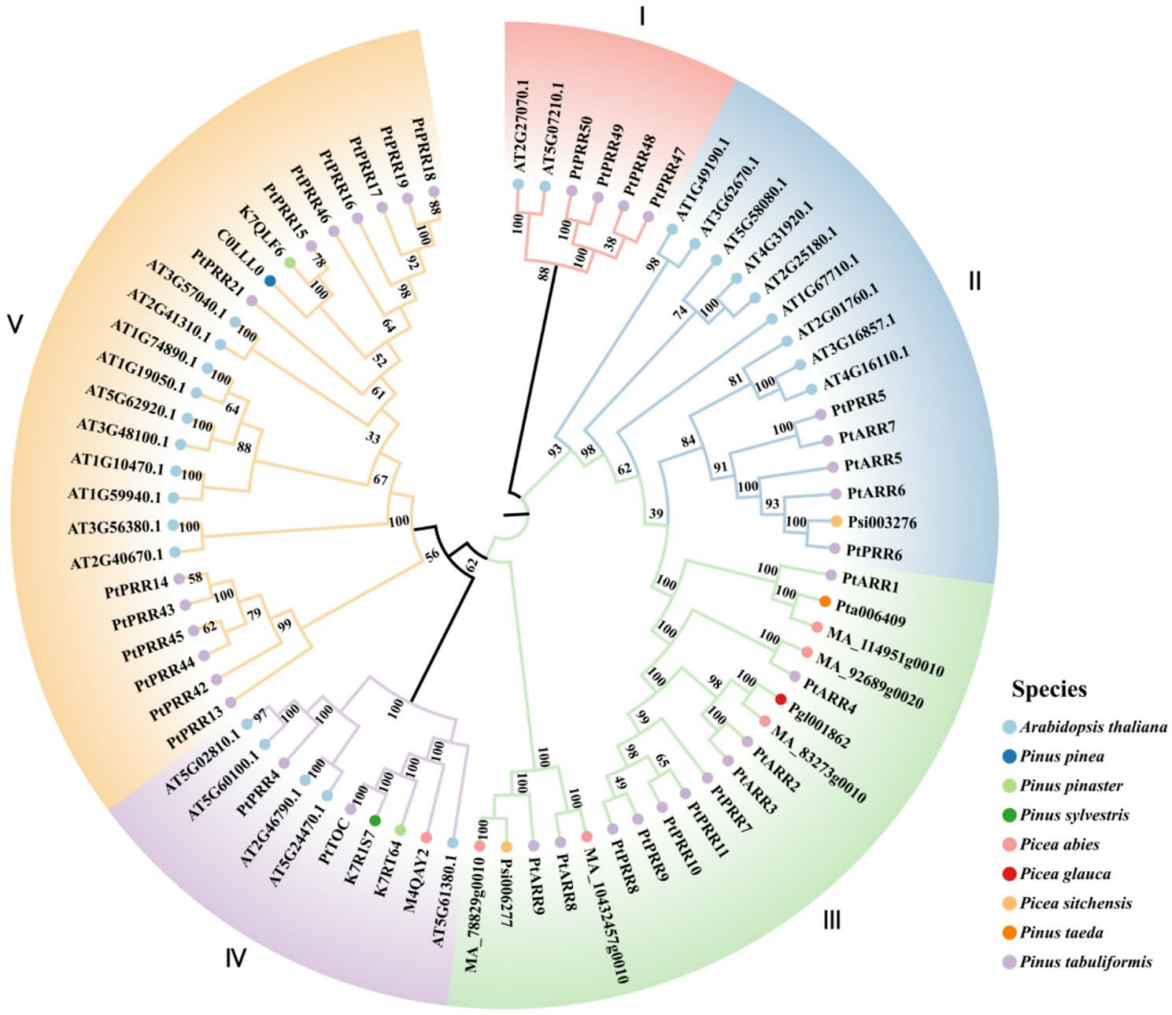
Phylogenetic tree of RR proteins from P. tabuliformis and various species. (This phylogenetic tree includes RR proteins from the model angiosperm A. thaliana and a selection of RR proteins from seven conifer species: P. pinea, Pinus pinaster, Pinus sylvestris, Picea abies, Picea glauca, Picea sitchensis, and Pinus taeda)

Phylogenetic analysis grouped the *PtRR* family into five distinct clusters (Fig. 2). Most A-ARR genes clustered within Group V, while *PtPRR47–50* formed a distinct cluster in Group I. Although these four genes clustered closely with two B-ARR genes, their lack of DNA-binding domains and high sequence similarity to other A-ARRs in BLAST analysis confirmed their classification as A-ARR members. Groups II and III encompassed the 16 B-ARR genes, while Group IV contained the two Pseudo-RR genes. Both the B-ARR and Pseudo-RR subfamilies exhibited a high degree of sequence conservation, emphasizing their evolutionary stability. The *P. tabuliformis PtTOC* gene showed high sequence similarity (100% bootstrap value) to *PRR1* genes from *Pinus pinaster*, *Pinus sylvestris*, and *Picea abies*, clustering closely with *AtTOC1*. This strong conservation suggests that conifer *TOC* proteins share common ancestral origins and functional roles with *AtTOC1*. Overall, the relationships among *PtRR* family members are consistent with our domain-based identification, underscoring the robustness of this classification framework.

### Gene structures and motif of PtRR genes

**Fig. 3:**
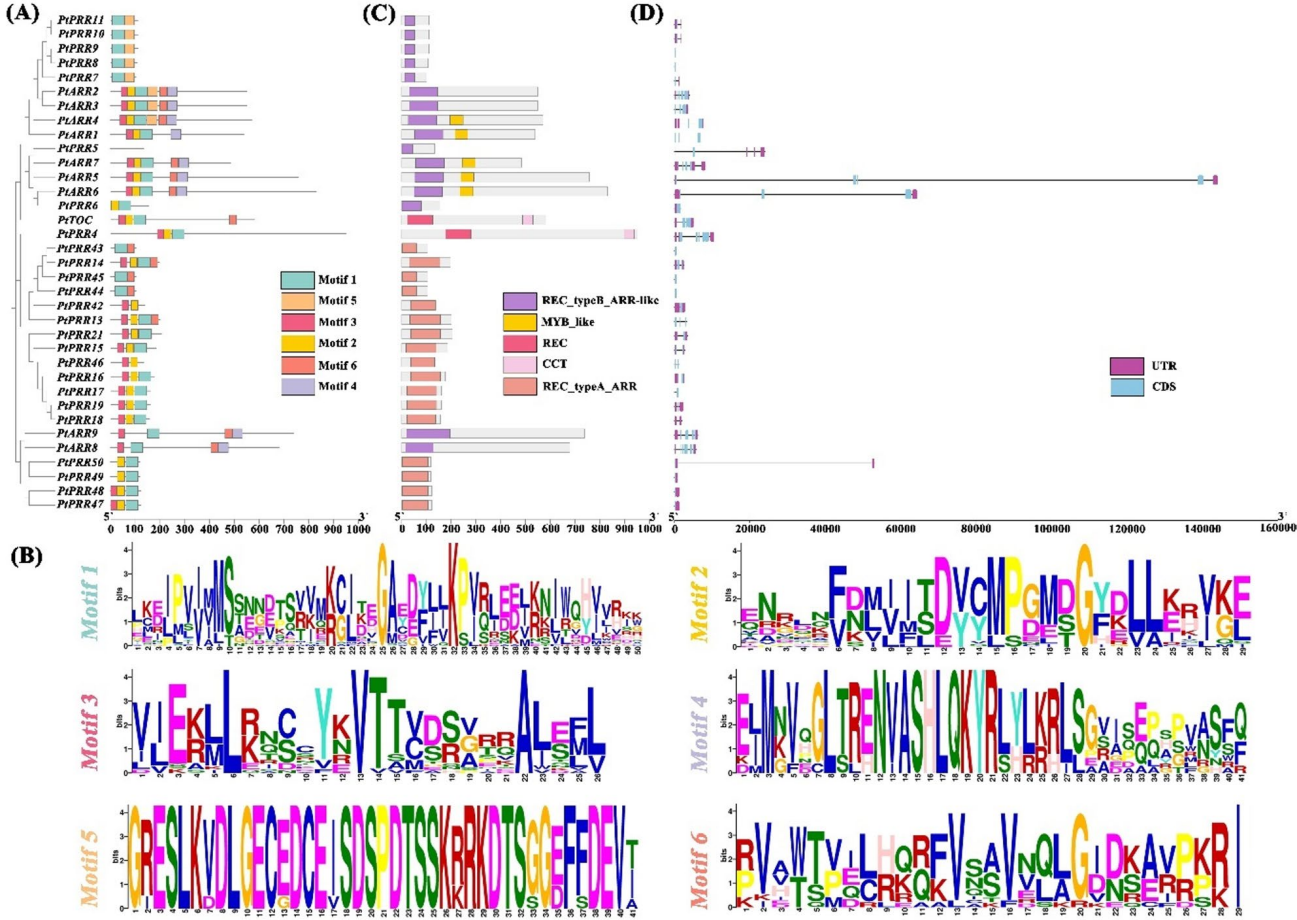
Distribution of protein structures within the RR gene family of *P. tabuliformis. ***A** Phylogenetic tree of the RR gene family alongside the MEME server analysis for predicting conserved motifs within the RR proteins. A total of six conserved motifs were identified (the scale at the bottom of each panel indicates the length of the protein and nucleotide sequences for each RR family member). **B **Word cloud representation of the six motifs identified in A, with the motif names corresponding to the colors used in the tree. **C** Distribution characteristics of the conserved domains within the RR gene family. **D** Structural organization of exons and introns within the RR genes

Phylogenetic analysis confirmed the evolutionary conservation of the A-ARR and B-ARR subfamilies, categorizing the *PtRR* gene family into four distinct sub-branches (Fig. 3). Notably, all genes, except for *PtPRR5*,* PtPRR42*, and *PtPRR46*, contain Motif 1, emphasizing its significance as a fundamental RR domain with key functional roles. Evolutionary Branch I consists of 14 B-ARR genes, where Motifs 5 and 2 are the second and third most prevalent motifs, respectively. Within this branch, *PtARR2*, *PtARR3*, and *PtARR4* are characterized by both Motifs 5 and 2, along with additional motifs. Branch II is unique in that it contains the only two Pseudo-RR genes, both of which include Motifs 1–3. Notably, *PtTOC* in this branch also features Motif 6. Branch III is primarily composed of A-ARR genes, though four A-ARR genes (*PtPRR47*,* PtPRR48*,* PtPRR49*, and *PtPRR50*) are grouped in a single cluster. This grouping aligns with previous classifications and is characterized by 2–4 motifs. The conserved exon-intron structures are closely correlate with these evolutionary branches, with A-ARR genes maintaining remarkably similar gene architectures across evolutionary time. In contrast, B-ARR genes exhibit more complex gene structures. Notably, *PtPRR5*,* PtARR5*,* PtARR6*, and *PtPRR50* have longer gene lengths and higher intron counts, with *PtARR5* being the longest. Recent genomic studies on *P. tabuliformis* support these findings, revealing a significant correlation between intron/exon length ratios and gene sizes across species, likely due to genomic expansions within these genes [29]. In contrast to the findings in *Medicago sativa*, where both A-type and B-type RRs contain untranslated regions (UTRs) [37], no UTRs have been identified in the pseudo-regulatory factor subfamily. However, both Pseudo-RR genes in *P. tabuliformis* contain more than two UTRs.

### Chromosomal distribution, synteny, and cis-regulatory elements analysis of PtRR genes

**Fig. 4:**
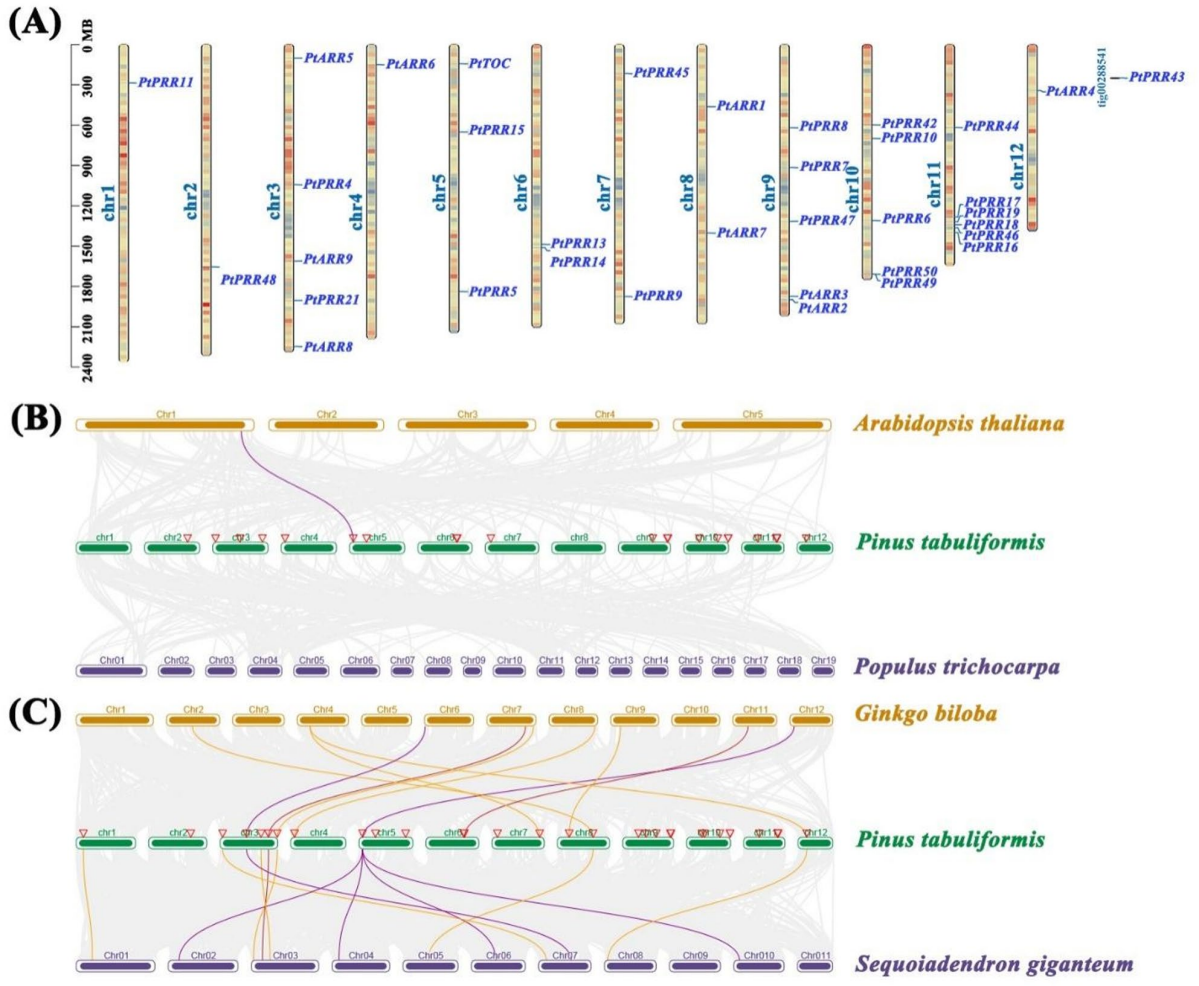
Chromosomal distribution of RR genes in P. tabuliformis and comparative synteny analysis across species. **A** A total of 34 RR family genes are localized on 12 chromosomes of P. tabuliformis, with the exception of PtPRR43. The scale on the left represents the physical length of the chromosomes; Mb = megabase pairs. **B** Comparative genomic synteny analysis of RR genes between P. tabuliformis and angiosperms (A. thaliana and P. trichocarpa), where the purple lines denote homologous relationships among the RR genes, and the gray lines indicate syntenic relationships across different species' genomes. Chromosomal bars and names for each plant are distinctly color-coded. **C** Comparative genomic synteny analysis of RR genes between P. tabuliformis and gymnosperms (G. biloba and S. giganteum), where the red lines represent homologous relationships of A-ARR subfamily genes, the orange lines denote homologous relationships of B-ARR subfamily genes, and the purple lines indicate homologous relationships of Pseudo-RR subfamily genes. The gray lines illustrate syntenic relationships across different species' genomes, with each plant's chromosomal bars and names similarly color-coded

Members of the RR gene family in *P. tabuliformis* were widely distributed across all chromosomes, with the exception of *PtPRR43*, which is located on an unassembled scaffold (Fig. 4A). Chromosomes 3, 9, 10, and 11 showed the highest densities of RR genes, with a particularly dense cluster on chromosome 11. This clustering may indicate local gene amplification or chromosomal rearrangements, which could have contributed to the diversification and functional differentiation of RR genes during evolution. Genes residing on the same chromosome are likely to interact synergistically, Participating in shared regulatory networks. To further explore the syntenic relationship of RR genes across different species and explore the evolutionary trajectory of the RR gene family, we generated synteny maps comparing *P. tabuliformis* with angiosperms such as *A. thaliana* and *P. trichocarpa*, as well as gymnosperms like *Ginkgo biloba* and *Sequoia giganteum*. Notably, *P. tabuliformis* does not exhibit any orthologs with *Populu*s, despite both being woody species. However, a single orthologous gene pair, *At1G74890* and *PtTOC*, was identified between *P. tabuliformis* and *A. thaliana* (Fig. 4B, Table S7). In contrast, 10 syntenic gene pairs were found with *G. biloba* and 12 with *S. giganteum* (Fig. 4C, Table S7). This pattern suggests as closer evolutionary relationship between *G. biloba*, *S. giganteum*, and *P. tabuliformis*, with the higher number of syntenic gene pairs observed with *S. giganteum* reflecting their classification within Conifer II, while *G. biloba* belongs to the Ginkgoales. Among the 22 syntenic gene pairs identified, B-ARR genes accounted for 50% (11 pairs), while Pseudo-RR genes, despite being limited member, represented 36.36% (8 pairs). In the synteny analysis with Sequoia, one-to-four syntenic relationships were observed, including those between *PtTOC* and *SEGI_16277*, *SEGI_11051*, *SEGI_03088*, and *SEGI_39684*. Overall, these findings suggest that B-ARR and Pseudo-RR subfamilies exhibit greater evolutionary conservation compared to the A-ARR subfamily.

**Fig. 5:**
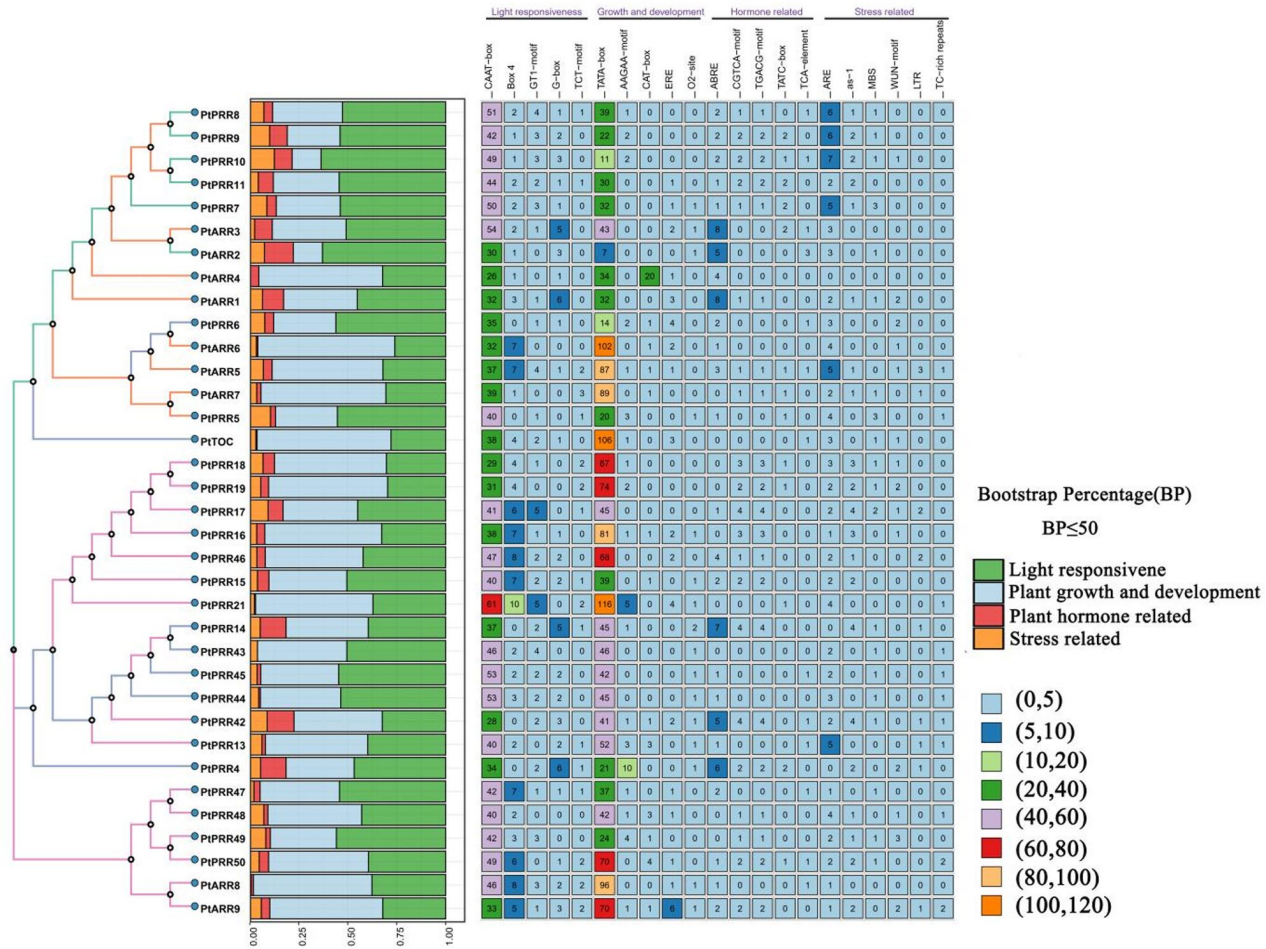
Analysis of cis-acting elements in RR genes of *P. tabuliformis. *(The constructed phylogenetic tree is consistent with that presented in Fig. 3. The left panel displays the statistical analysis of the percentage and number of four distinct classes of cis-regulatory elements. The right panel illustrates the predicted distribution and number of various cis-regulatory elements across the promoter sequences of different RR genes, which include five types associated with light responsiveness, five types related to growth and development, five types involved in hormone, and six types linked to stress)

Promoters of the RR genes in *P. tabuliformis* reveals a high abundance of cis-regulatory elements associated with plant growth and development (Fig. 5). Each RR gene promoter contains at least 11 TATA-box motifs, with *PtPRR21* and *PtTOC* exhibiting the highest counts—116 and 106 motifs, respectively. These findings further support the crucial role of the RR gene family in regulating plant growth and developmental processes. Additionally, light-responsive regulatory elements are highly enriched in the promotors of these genes, emphasizing the importance of photoperiod regulation in controlling the plant circadian clock. This regulation plays a key role in growth, development, and flowering time. Photoperiodic pathways are highly conserved across seed plants, as evidenced by the *P. tabuliformis* genome project. Nearly all RR gene promoters contain multiple copies of light-responsive elements, suggesting these genes likely modulate growth and development through light-mediated pathways. The most prominent light-responsive motifs include the CAAT-box, Box 4, GT1, and G-box. Among these, the G-box motif is particularly crucial for the plant’s response to photoperiods, as it regulates the timing of flowering genes expression under the control of *TOC1*. In addition to growth and development, promoters of genes such as *PtARR1*, *PtARR2*, and *PtARR3* are enriched with ABRE (abscisic acid-responsive elements), suggesting that these genes may play dual roles cytokinin and abscisic acid signaling. Although RR genes are also involved in stress-related signaling, these elements are less abundant in the promoters compared to those associated with growth and development, indicating that the primary function of these genes is in regulating physiological processes linked to plant growth and development, and environmental responses.

### **Expression profiling of PtRR genes in *P. tabuliformis***

**Fig. 6:**
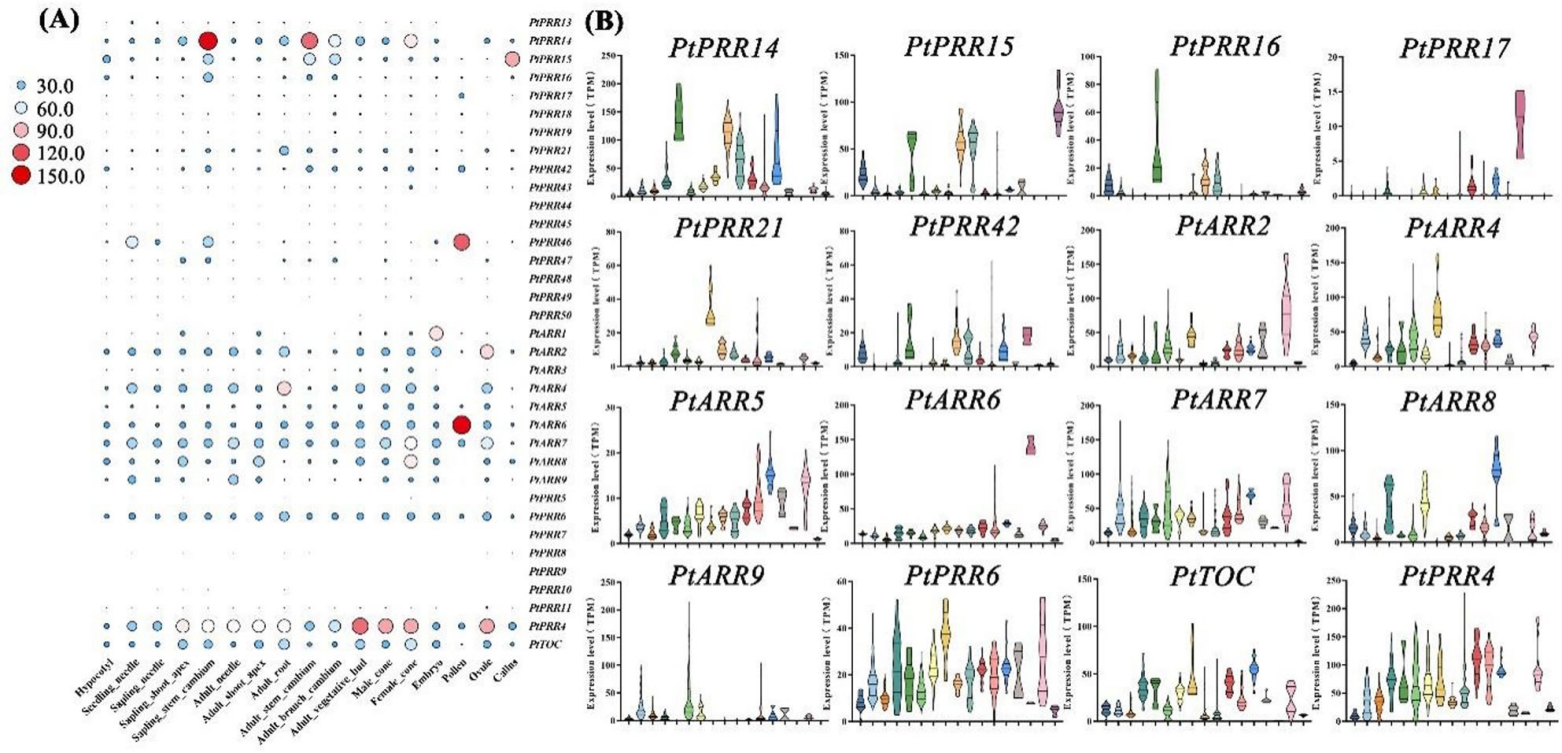
Expression analysis of RR genes in various organs of *P. tabuliformis. ***A **Heatmap illustrating the expression levels of RR genes across different organs, where the circle size increases and the color gradient transitions from blue to red, indicating expression levels from low to high. **B **Expression profiles of 16 RR genes, which exhibit broad expression across various organ tissues in *P. tabuliformis. *The analyzed samples included: hypocotyls (n = 36), seedling needles (n = 90), sapling needles (n = 165), Sapling shoot apex (n = 51), Sapling stem cambium (n = 51), Adult needle(n = 468), Adult shoot apex (n = 69), Adult root (n = 16), Adult stem cambium (n = 51), Adult branch cambium (n = 60), Adult vegetative bud (n = 21), Male cone (n = 60), Female cone (n = 18), Embryo (n = 6), Pollen (n = 3), Ovule (n = 24), Callus (n = 18)

Among 35 RR genes analyzed, 16 exhibited distinct yet widespread expression patterns across various tissues and developmental stages (Fig. 6A, B, Figure S1). Notably, A-ARR genes, such as *PtPRR14*,* PtPRR15*, and *PtPRR16*, showed elevated expression in the cambium of both seedlings and mature trees, as well as in branches. This suggests these genes play a key role in regulating growth and secondary development, likely by suppressing excessive cytokinin signaling. Such regulation ensures precise control of cell division and differentiation during both primary and secondary growth. *PtPRR46* and *PtARR6* were found to be highly expressed in pollen grains, where enhanced cytokinin signaling is crucial for promoting cell division, expansion, and tissue morphogenesis. Given that A-ARR genes are known to be targets of B-ARR genes, their potential synergistic interactions may contribute to pollen maturation and vitality, thus supporting reproductive success in *P. tabuliformis* in response to environmental stimuli. Additionally, *PtPRR4* exhibited increased expression in ovules and both male and female cones, suggesting a significant role in the development of reproductive structures. In contrast to the broader expression profiles of in A-ARR and B-ARR genes, *PtTOC* displayed a more restricted expression profile across different organs. This suggests that *PtTOC* may have a specialized function, likely related to environmental adaptability, particularly in regulating circadian rhythms and photoperiodic responses, rather than directly controlling growth.

### Diurnal and annual oscillation of *PtTOC* expression under the influence of photoperiod and temperature

**Fig. 7:**
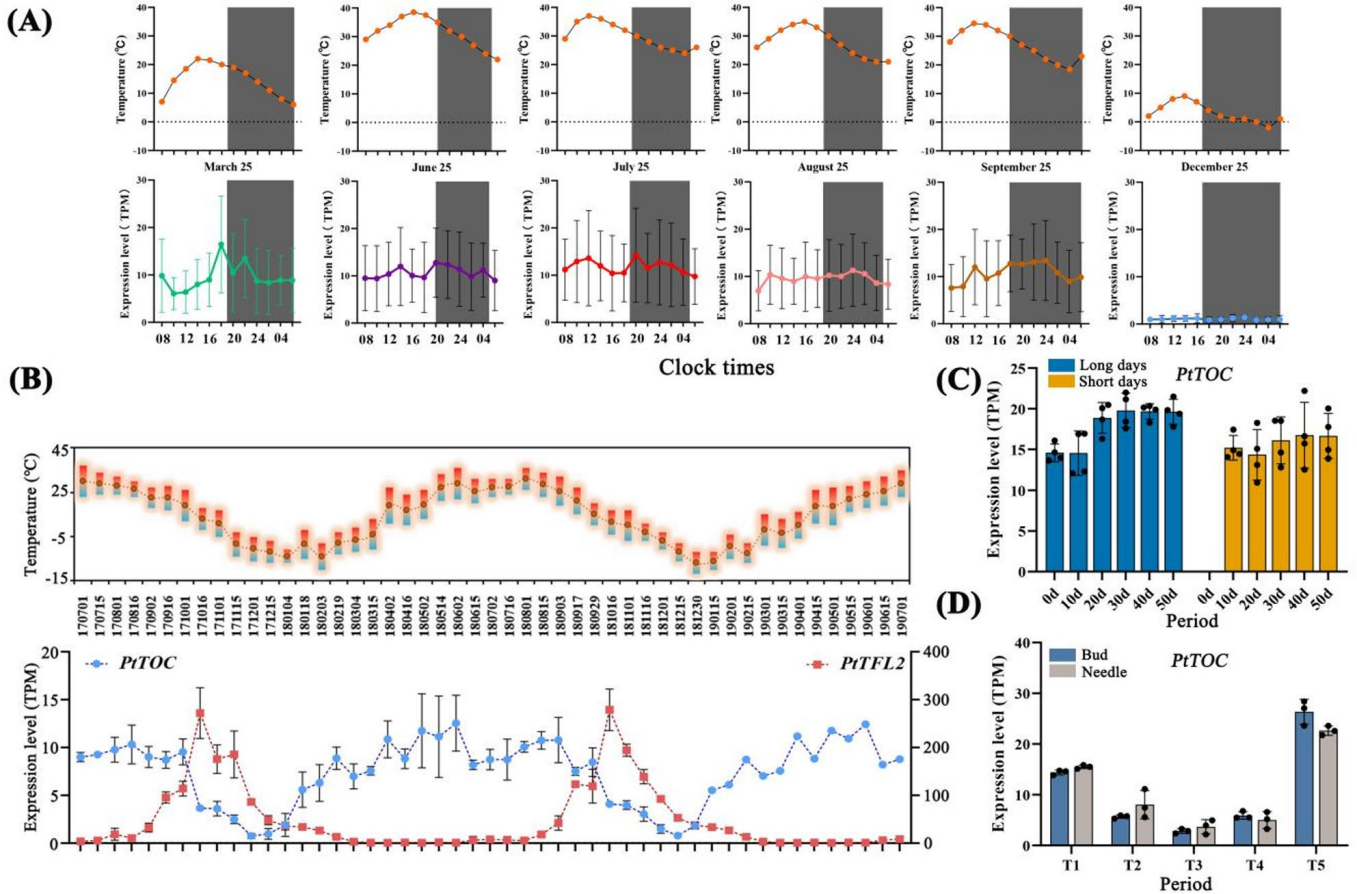
Expression profiles of *PtTOC* across various temporal scales and environmental conditions. **A **Expression levels of PtTOC at 8:00, 12:00, 16:00, 20:00, 24:00, and 4:00 on March 25, June 25, July 25, August 25, September 25, and December 25. Error bars indicate the variability observed across three independent replicates. **B** Annual expression profiles of PtTOC and PtTFL2 in the needles of P. tabuliformis. Monitoring was conducted over two years, from July 1, 2017, to July 1, 2019. Error bars reflect variability from three independent replicates. **C** Variations in the expression levels of PtTOC in the needles of P. tabuliformis under different photoperiod conditions (long days: light: dark, 16:8 h, 24℃, short days: light: dark, 8: 16 h, 24℃). **D** Changes in the expression of PtTOC in the needles and apical buds of P. tabuliformis under simulated winter conditions. T1: Treatment under short-day warm conditions (light: dark, 8: 16 h, 24 °C) for 8 weeks; T2-T4: Continuous treatment under short-day cold conditions (light: dark, 8: 16 h, 4°) for 1, 3, and 7 weeks, respectively; T5: Treatment under long-day warm conditions (light: dark, 16:8 h, 24 °C) for 2 weeks

Distinct oscillations in *PtTOC* expression were observed, with peaks in March and additional fluctuations occurring on June 25, July 25, August 25, and September 25 (Fig. 7A). The expression levels were lowest between 8:00 and 10:00 AM, reaching a peak between 6:00 and 8:00 PM, followed by a gradual decline throughout the night, establishing a clear circadian rhythm. Notably, *PtTOC* expression was nearly absent in December, suggesting that low temperatures may suppress its expression and indirectly affect the circadian cycle in *P. tabuliformis*. To further elucidate the role of *PtTOC* as a core oscillator, we assessed its annual expression profile in needles over two years using RNA-seq (Fig. 7B). Significant seasonal oscillations were observed, with expression levels peaking in May and June during the the summer, followed by a gradual decline from September to October and reaching a minimum in December. Expression then recovered as temperature rose. This pattern underscores the crucial role of *PtTOC* in regulating the circadian rhythm of *P. tabuliformis* and highlights the influence of temperature on its transcriptional abundance. Additionally, *PtTOC* expression was modulated by photoperiod, with long-day conditions promoting higher expression levels in needles compared to short-day conditions (Fig. 7C). Low temperatures further inhibited *PtTOC* expression. As illustrated in Fig. 7D, transitioning *P. tabuliformis* from short-day ambient to short-day low-temperature conditions resulted in a rapid decline in *PtTOC* expression in needles and shoot buds. Conversely, exposure to long-day ambient conditions led to a nearly a five-fold increase in *PtTOC* expression. These findings indicate that both short photoperiods and low temperatures suppress *PtTOC* expression, influencing diurnal and annual oscillations in *P. tabuliformis*.

### Dual repression yields activation effect: *PtTOC* promotes flowering by negatively regulating the floral repressor *PtTFL2*

**Fig. 8:**
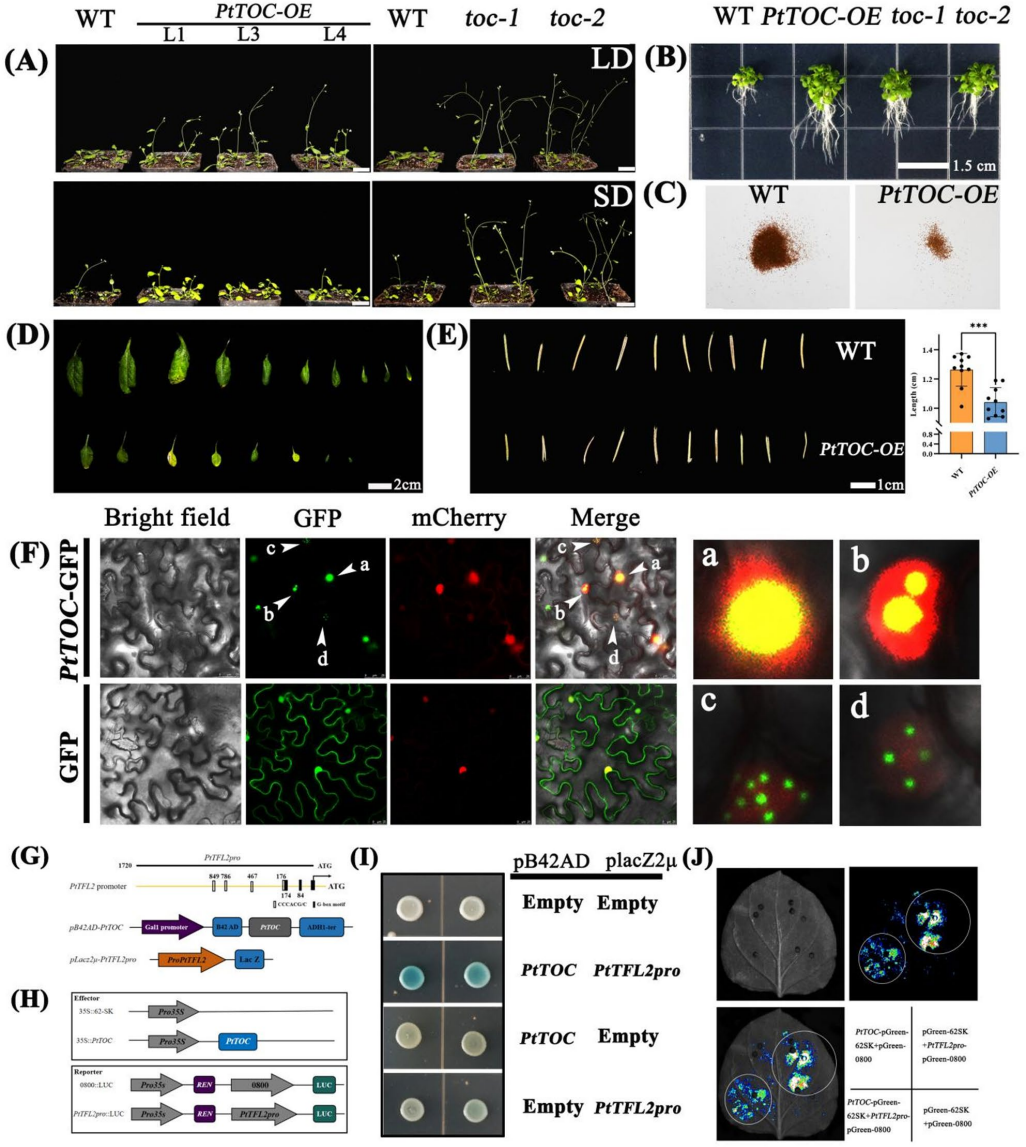
PtTOC promotes early flowering by inhibiting the transcription level of PtTFL2. **A** After two weeks of growth under long-day conditions, three transgenic A. thaliana lines (PtTOC-OE-1/3/4) and the toc1/2 mutants exhibited phenotypes indicative of early flowering. In contrast, following three weeks of growth under short-day conditions, the toc1 and toc2 mutants also displayed early flowering phenotypes, whereas the early flowering phenotype in PtTOC-OE was less pronounced. **B** On MS medium, following one week of growth under standard conditions, both the transgenic A. thaliana PtTOC-OE and the toc1/2 mutants demonstrated enhanced root growth **C** The seed yield of transgenic A. thaliana PtTOC-OE was notably lower than that of the wild type (WT). **D **Rosette leaves of transgenic A. thaliana PtTOC-OE were not only smaller in size but also fewer in number compared to those of the WT. **E **Length of pods in transgenic A. thaliana PtTOC-OE was significantly reduced relative to the WT. **F** Subcellular localization of GFP and PtTOC-GFP: panels a, b, c, and d depict various morphological changes of the PtTOC-GFP protein during subcellular localization, with the scale bar indicating 25 μm. **G** Schematic representation of the pB42AD-PtTOC and pLacz2μ-PtTFL2pro promoter constructs employed in the Y1H assay. **H** Schematic diagram of the effector and reporter gene constructs for PtTOC and PtTFL2pro promoters. **I** Y1H assay demonstrating the direct binding of PtTOC to the PtTFL2pro promoter, involving yeast cells co-transformed with pB42AD-PtTOC/pLacz2μ-PtTFL2pro and cultured on selective medium (SD/−Trp/-Ura + Gal+Raf+X-Gal). pLacZ2μ/pB42AD, pLacZ2μ/pB42AD-PtTOC, and pLacz2μ-PtTFL2pro/pB42AD were utilized as negative controls. **J** Interaction between PtTOC and PtTFL2pro promoters in N. benthamiana leaves was assessed using a luciferase (LUC) assay

Compared to wild-type *A. thaliana* (*Col-0*), the *PtTOC* overexpression (*OE*) lines exhibited accelerated flowering under long-day conditions, consistent with transcriptomic data showing elevated *PtTOC* expression in these conditions (Figs. 7C and 8A). However, this early flowering phenotype was not observed under short-day conditions. *PtTOC* overexpression also impacted root morphology, resulting in longer roots, fewer and smaller rosette leaves, and smaller siliques with reduced seed yield compared to *Col-0* (Fig. 8B-E). Interestingly, the *toc-1* and *toc-2* mutants of *A. thaliana* flowered early under both long- and short-day conditions (Fig. 8A), suggesting that floral repressors may mitigate the flowering-promoting effects of *PtTOC* under short-day conditions. The *TFL-like* genes, which act antagonistically to flowering signals and are partially downstream of *CO*, are known to facilitate early flowering when absent [38]. Transcript levels of the floral repressor *PtTFL2* increased under short-day conditions [39, 40], exhibiting an inverse annual expression pattern relative to *PtTOC.* This suggests a potential regulatory relationship between *PtTOC* and *PtTFL2* (Fig. 7B). While these heterologous expression results in *A. thaliana* provide valuable functional insights, the distinct regulatory landscape of gymnosperms necessitates a cautious extrapolation of these findings to *P. tabuliformis*. A comprehensive discussion regarding the limitations of this heterologous expression system is provided in the Discussion section.

Subcellular localization studies revealed that *PtTOC* is predominantly nuclear, supporting its role as a transcriptional regulator (Fig. 8F). Notably, *PtTOC* proteins displayed dynamic forms within the nucleus, suggesting possible protein phase transitions. The CCAC motif is essential for *TOC1* recognition in both *A. thaliana* and *O. sativa* [9]. Although *AtTOC1*-binding motifs (CCACAC) were not identified in the 2-kb upstream region of the *PtTFL2* promoter, two G-box motifs (CACGTG/C) and four *OsTOC1*-binding motifs (G/CCCACG/C) were detected (Fig. 8G). To further investigate interactions between *PtTOC* and the *PtTFL2* promoter, Y1H and dual-luciferase assays were performed (Fig. 8G, H). Y1H assays demonstrated direct interactions between *PtTOC* and the *PtTFL2* promoter fragment, activating its expression, as indicated by yeast turning blue (Fig. 8I). As expected, transient transcription assays in *N. benthamiana* revealed that co-transformation of *PtTOC*-pGreen 62SK with *PtTFL2pro*-pGreen 0800 led to a significant decrease in luciferase activity compared to the control vector, confirming that *PtTOC* represses *PtTFL2* transcription (Fig. 8J, Figure S2). These findings suggest that *P. tabuliformis* can directly regulate *PtTFL2*-mediated flowering time through the core circadian oscillator gene *PtTOC*.

### *PtTOC* overexpression compromises freezing tolerance while enhancing heat resilience in plants

Short photoperiods and low temperatures significantly influence *PtTOC* expression (Fig. 7). It is well-documented that *TOC1*, along with several members of the pseudo-regulator subfamily, act as a negative regulator, repressing the transcription of cold-responsive genes and their downstream targets [19, 41]. Recently, *PtTFL2* was shown to interact with *PtNAC67*, forming a protein complex that enhances freezing tolerance [42]. Therefore, the inhibitory effect of *PtTOC* on *PtTFL2* transcription may contribute to the modulation of plant freezing resistance.

**Fig. 9:**
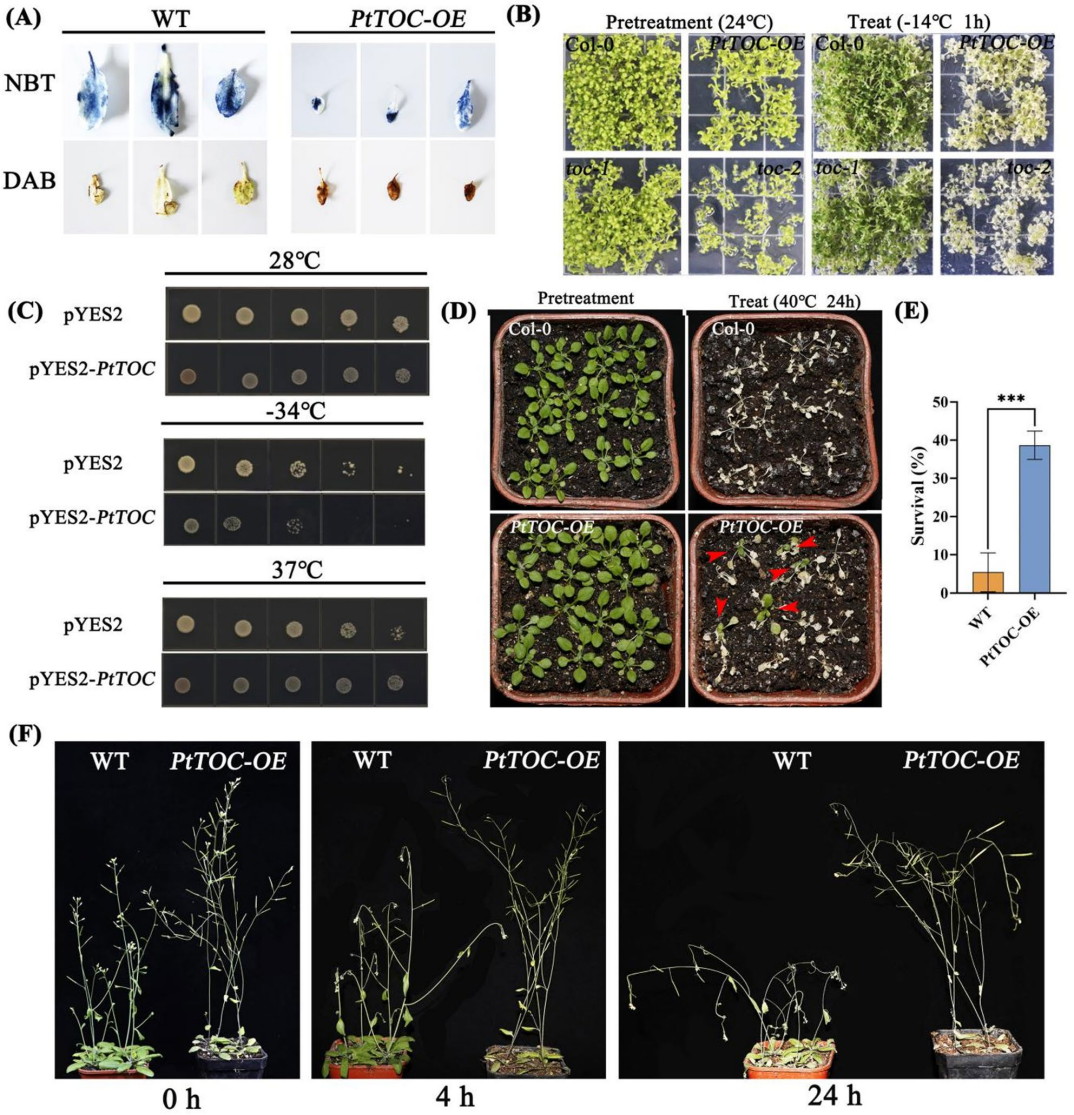
Histological staining of *PtTOC*, analysis of transgenic *A. thaliana* resistance, and yeast resistance assessment. **A** NBT and DAB staining of leaves from *Col-0* (wild type) and *PtTOC-OE* under both non-stress and cold stress conditions; deeper coloration indicates a higher degree of stress-induced damage in *A. thaliana*. **B** Phenotypes of *Col-0* and *PtTOC-OE* before and after cold treatment. **C** Growth assessment of yeast strains harboring pYES2-*PtTOC* and pYES2 under various temperature conditions. **D**-**F** Phenotypes and surivial of Col-0 and *PtTOC-OE* before and after heat stress treatment

Histological staining revealed that after two weeks at 4 °C, *PtTOC-OE* plants accumulated significantly higher levels of superoxide (O₂⁻) and hydrogen peroxide (H₂O₂) compared to wild-type (WT) plants, indicating enhanced oxidative stress in the *PtTOC-OE* lines (Fig. 9A). Furthermore, under freezing stress at − 14 °C, *PtTOC-OE* plants exhibited lower survival rates than WT plants, while the *toc1* mutant showed significantly higher freezing tolerance (Fig. 9B). A similar trend was observed in cold sensitivity assays using the BY4744 yeast strain: at 28℃, yeast cells carrying the Pyes2-*PtTOC* construct showed growth comparable to the empty vector control. However, under low-temperature stress at − 34℃, yeast carrying Pyes2-*PtTOC* exhibited slower growth than the control on SD-URA medium, suggesting that *PtTOC* impairs cold tolerance in yeast (Fig. 9C).

In contrast, *PtTOC-OE* plants demonstrated enhanced thermotolerance. When exposed to heat stress at 37℃, yeast expressing Pyes2-*PtTOC* grew faster than the empty vector control (Fig. 9C). Similarly, after 24 h at 40℃ in a growth chamber, *PtTOC-OE* plants exhibited higher survival rate than WT plants and maintained better overall growth under heat stress (Fig. 9D and F). These results collectively indicate that *PtTOC* positively regulates heat stress tolerance.

## Discussion

This study delivers a comprehensive genome-wide analysis of the RR gene family in *Pinus tabuliformis*, providing the most detailed picture to date of RR evolution, expression dynamics, and physiological function in a gymnosperm. Although advances in sequencing technology have enabled systematic RR analyses across diverse angiosperm lineages — ranging from model herbs to woody crops [13, 23–26, 43]— gymnosperms have remained a conspicuous gap in our understanding of plant two-component signaling. By integrating phylogenomics, expression profiling, and molecular functional assays, we establish that *P. tabuliformis* harbors 35 RR genes distributed across three subfamilies — 17 A-ARRs, 16 B-ARRs, and 2 pseudo-RRs — a complement that exceeds the count in *A. thaliana* and is broadly comparable to that of *P. trichocarpa* and *O. sativa* [23]. Critically, this numerical similarity conceals a striking compositional divergence: whereas angiosperms have expanded their pseudo-RR repertoire to five or more members that collectively tile the circadian day, *P. tabuliformis* retains only 2 pseudo-RR genes. This divergence is particularly intriguing when considering the larger genome size of *P. tabuliformis*, which contains approximately 82,000 genes—1.8 to 3 times larger than the genomes of *P. trichocarpa* (~ 45,000), *O. sativa* (~ 41,000), and *A. thaliana* (~ 27,000) [29, 44–47]. The disproportionate underrepresentation of pseudo-RRs relative to overall genome size argues that their expansion in angiosperms reflects lineage-specific selection pressures — perhaps related to the diversification of photoperiodic flowering pathways — rather than a universal consequence of genome size or complexity. In contrast, the pronounced expansion of A- and B-ARR subfamilies in *P. tabuliformis* repositions these classical cytokinin signaling components as potentially dominant players in gymnosperm developmental regulation, and invites a broader reconsideration of how the HK–HP–RR phosphorelay pathway has been rewired across the seed plant phylogeny [48–50] .

Beyond their phylogenetic distribution, the expression profiles of A- and B-ARRs in *P. tabuliformis* reveal tissue-specific partitioning that is broadly conserved with angiosperm counterparts, yet adapted to the distinctive developmental architecture of conifers. A-ARR genes — notably *PtPRR14*, *PtPRR15*, and *PtPRR16* — are preferentially expressed in the cambium of both juvenile seedlings and mature trees, consistent with their proposed role as negative feedback regulators that fine-tune cytokinin signaling during secondary growth and vascular differentiation. This pattern mirrors the elevated vascular expression documented for Populus ARR homologs [23], underscoring the conservation of A-ARR function in wood-forming tissues across divergent woody lineages. B-ARRs exhibit a complementary expression pattern, with *PtARR2*, *PtARR4*, and *PtARR7* showing comparatively higher levels in needles, shoot buds, and roots — tissues characterized by active cell proliferation, meristematic activity, or responses to environmental stimuli. This preference is consistent with the well-established role of B-type ARRs as transcriptional activators in the cytokinin signaling cascade: in *A. thaliana*, *ARR1* promotes root elongation by upregulating auxin biosynthesis genes [51], and collaborates with *ARR12* to sustain stem cell homeostasis in the shoot apical meristem [52]. In the reproductive context — a domain of particular significance for monoecious Pinaceae species whose reproductive biology has historically received limited molecular attention — the pseudo-RR gene *PtPRR4* displays notably elevated expression in ovules and in both male and female cones. This organ-enriched expression pattern positions *PtPRR4* as a candidate regulator of reproductive organ morphogenesis and differentiation, and marks it as a priority target for future functional dissection of cone development abnormalities in gymnosperms. Collectively, these expression data suggest that the functional subdivision of the RR gene family into growth-regulatory (A/B-ARR) and environmental-sensing (pseudo-RR) modules, which is well-established in angiosperms, has been preserved in conifers but further elaborated through lineage-specific gene expansion and subfunctionalization.

The core circadian clock in angiosperms is organized around an interlocking transcription—translation feedback loop in which the evening-phased repressor *TOC1* suppresses the morning-phased activators *CCA1* and *LHY*, which in turn repress *TOC1* transcription at dawn to complete the ~ 24-hour oscillation [8, 13]. Our transcriptomic analysis of *PtTOC* across multiple temporal scales provides the first evidence that this fundamental oscillator architecture is preserved in *P. tabuliformis*, albeit with intriguing modifications imposed by the conifer’s perennial lifestyle. At the diurnal scale, *PtTOC* expression reaches a nadir between 08:00 and 10:00 and peaks between 18:00 and 20:00 — an evening-phased rhythm that closely mirrors *TOC1* dynamics in *A. thaliana* [8] and is consistent with the co-oscillatory behavior of the putative *PtCCA1* and *PtLHY*-like genes (*PtMYB266*, *PtMYB267*, and *PtMYB270*) observed in our two-year transcriptome dataset (Figures S3 and S4). While the mutual inhibitory relationship between *PtCCA1/LHY*-like genes and *PtTOC* awaits direct experimental validation in *P. tabuliformis*, the anti-phasic annual expression profiles of these genes — with *PtCCA1*-like genes peaking during winter dormancy while *PtTOC* is nearly absent in December — strongly imply the existence of an analogous feedback mechanism. At the seasonal scale, *PtTOC* expression exhibits pronounced peaks in May–June, coinciding with the onset of pollen shedding and female cone receptivity, and declines sharply as temperatures fall in autumn, reaching a nadir in December when daily minimum temperatures regularly fall below 0 °C at the species’ primary distribution range in northern China. The near-complete transcriptional silence of *PtTOC* during winter, combined with its rapid reinduction following the transition to long-day warm conditions (a nearly five-fold increase; Fig. 7D), identifies temperature as the dominant environmental input governing *PtTOC* abundance — a conclusion further supported by the observation that low-temperature stress suppresses *PtTOC* expression even under long-day conditions that would otherwise sustain its transcription. This temperature-gated seasonal rhythm provides a compelling molecular framework for understanding how *P. tabuliformis* synchronizes its reproductive development with the thermal seasons, and positions *PtTOC* as a key node at which circadian timekeeping and thermosensory signaling converge.

Flowering time in conifers like *P. tabuliformis* is strongly influenced by temperature. Unlike deciduous species, pines retain their needles during dormancy, making them highly responsive to environmental cues. The male cone buds are protected by bud scales, but still, they are influenced by environmental factors, particularly temperature [53]. In *A. thaliana*, the gene *TOC1* regulates the circadian clock and influences flowering time by modulating rhythmic patterns [54]. The functional characterization of *PtTOC* in the heterologous *A. thaliana* system yielded a series of phenotypes that, taken together, support a model in which *PtTOC* promotes floral transition through a double-negative regulatory mechanism: as a transcriptional repressor that directly suppresses the floral repressor *PtTFL2*, *PtTOC* effectively derepresses the flowering program in a manner that is contingent on both photoperiod and temperature. Under long-day conditions, *PtTOC*-OE lines flowered significantly earlier than wild-type *Col-0* plants, whereas this acceleration was largely absent under short-day conditions — a photoperiod-specific response that recapitulates the long-day-induced accumulation of *PtTOC* observed in pine needles (Fig. 7C). The molecular basis of this regulation was elucidated through complementary biochemical approaches: Y1H assays confirmed direct physical interaction between *PtTOC* and the *PtTFL2* promoter, while dual-luciferase reporter assays in Nicotiana benthamiana demonstrated that *PtTOC* co-expression significantly represses *PtTFL2pro*-driven transcription relative to vector controls. Notably, although the canonical *AtTOC1*-binding motif (CCACAC) was absent from the 2-kb *PtTFL2* promoter region, four *OsTOC1*-binding motifs (G/CCCACG/C) and two G-box elements (CACGTG/C) were identified — an observation that suggests *PtTOC* has retained DNA-binding specificity more closely related to the rice *OsTOC1* than to *AtTOC1*, consistent with the deep divergence of gymnosperm circadian regulatory circuits from the *Arabidopsis* model. This double-negative logic — repressor inhibiting repressor to yield net activation — has elegant precedent in angiosperm flowering regulation, where *TOC1* suppresses CDF proteins that would otherwise repress *CO*, thereby permitting *CO-*driven *FT* activation [17–19, 55]. In gymnosperms, which lack a bona fide FT ortholog, our data suggest that a parallel but distinct circuit operates, in which *PtTOC*-mediated suppression of *PtTFL2* serves as the functional equivalent, enabling floral transition to proceed when environmental conditions are permissive. The broader significance of this finding lies in its implication that the double-negative architecture of flowering regulation — wherein a circadian repressor gates a floral brake — predates the angiosperm–gymnosperm divergence and represents an ancestral regulatory logic conserved across seed plant lineages, even as the specific molecular players have diversified. Additional regulatory complexity is provided by the potential involvement of *NUCLEAR FACTOR Y* (*NF-Y*) subunits: in *A. thaliana*, *NF-YB* and *NF-YC* form a trimeric complex with *TOC1* that stabilizes its binding to target gene promoters and recruits *HISTONE DEACETYLASE 15* to repress transcription [14]. Whether a cognate *NF-Y–PtTOC* complex operates at the *PtTFL2* promoter in *P. tabuliformis* remains an open question that warrants investigation through co-immunoprecipitation and chromatin immunoprecipitation approaches.

While the heterologous experimental framework employed here has generated mechanistically coherent and internally consistent insights, the extrapolation of functional conclusions from *A. thaliana* to *P. tabuliformis* requires explicit caution, given the profound evolutionary distance and distinct regulatory landscapes separating these two lineages. The gymnosperm–angiosperm divergence dates to approximately 300–350 million years ago, and the intervening period has witnessed the progressive elaboration of angiosperm-specific flowering pathways that are wholly absent in gymnosperms. Most consequentially, the TOC1–CO–FT regulatory cascade that lies at the heart of photoperiodic flowering control in *A. thaliana* [16, 17] has no direct equivalent in *P. tabuliformis*: comprehensive genomic surveys of *Pinus*, *Picea*, and *Ginkgo* have consistently failed to identify bona fide *FT* orthologs [21]. It follows that the early-flowering phenotype of *PtTOC-OE* plants in *A. thaliana* likely reflects the engagement of *PtTOC* with the angiosperm-specific TOC1→CO→FT pathway, and cannot be assumed to represent a faithful readout of *PtTOC* function in the native gymnosperm context. Rather, the direct biochemical evidence from Y1H and dual-luciferase assays — which demonstrate *PtTOC* binding to and repression of the *PtTFL2* promoter in a species-neutral cellular background — provides the most transferable mechanistic insight. A second important caveat concerns the protein interaction landscape. In *A. thaliana*, *TOC1* protein stability is post-translationally controlled by the blue-light-dependent F-box proteins ZTL and FKF1, which mediate proteasomal degradation of *TOC1* during the day and thereby contribute to its evening-phased accumulation. While *ZTL/FKF1*-like sequences exist in the *P. tabuliformis* genome, their biochemical properties and diurnal dynamics in conifers remain entirely uncharacterized, raising the real possibility that *PtTOC* protein stability follows a divergent regulatory kinetics in its native cellular environment. Similarly, the *NF-YB/C* interaction interface that stabilizes *AtTOC1* at target promoters [14] may not be conserved in *P. tabuliformis*, where *NF-Y* subunit composition and target gene specificity are poorly understood. Overexpression of *PtTOC* under the constitutive 35 S promoter in *A. thaliana* bypasses all of these post-translational checkpoints and eliminates the temporal gating of *PtTOC* activity by the circadian clock itself, potentially generating phenotypic outcomes that are exaggerated or qualitatively different from those arising under native spatiotemporal expression patterns. These considerations collectively underscore that the present findings should be regarded as a mechanistically well-grounded hypothesis platform, rather than a definitive functional portrait of *PtTOC* in *P. tabuliformis*. Full validation in the native gymnosperm context will require the application of emerging conifer functional genomics tools — including VIGS approaches being developed for *Picea* and *Pinus*, protoplast transactivation systems, and the gymnosperm transformation platforms currently being optimized for Larix and other tractable conifer species — which will be essential to interrogate *PtTOC* function without the confounding influence of divergent angiosperm regulatory machinery.

The temperature-dependent regulation of *PtTOC* expression not only shapes its role in seasonal flowering but also positions it at the intersection of circadian timekeeping and cold stress adaptation — a dual function with important implications for understanding the ecological resilience of *P. tabuliformis* across its broad latitudinal distribution. Conifers exhibit sophisticated cold acclimation responses, in which the perception of shortening photoperiods and declining autumn temperatures jointly triggers growth cessation, bud set, and the induction of freezing tolerance [56]. Cold signaling in plants proceeds through both *C-REPEAT/DRE BINDING FACTOR* (*CBF*)-dependent and *CBF*-independent pathways [57], with *CBF* transcription factors serving as master regulators of cold-responsive gene expression [58]. The interconnection between the circadian clock and *CBF* regulation has emerged as a critical theme: positive regulators including *INDUCER OF CBF EXPRESSION* (ICE)*1/2* and *CCA1/LHY* enhance *CBF* transcription and downstream cold-responsive gene expression, thereby potentiating freezing tolerance, whereas *TOC1* acts as a negative regulator that suppresses *CBF* transcription and attenuates ABA signaling downstream of the receptor — an inhibitory function conserved across *A. thaliana* and rice [41, 59]. Our finding that *PtTOC-OE* plants accumulate elevated levels of O₂⁻ and H₂O₂ following cold treatment, coupled with their significantly reduced survival following − 14 °C freezing stress and the impaired cold tolerance of *PtTOC*-expressing yeast at − 34 °C, provides convergent evidence that *PtTOC* acts as a negative regulator of cold tolerance in conifers, likely through suppression of *CBF*-dependent transcriptional cascades. This interpretation is mechanistically coherent: the seasonal nadir of *PtTOC* expression in December — when freezing temperatures are most acute — would naturally relieve its inhibitory pressure on *CBF* genes, allowing maximal cold acclimation to proceed. Conversely, the peak of *PtTOC* expression in late spring and early summer, coinciding with reproductive development, would suppress cold-response pathways under conditions where they are unnecessary and metabolically costly. This reciprocal relationship between *PtTOC*-driven reproductive promotion and *CBF*-mediated cold tolerance constitutes an elegant seasonal trade-off: high *PtTOC* in warm seasons supports flowering by suppressing *PtTFL2* while simultaneously reducing the cold tolerance apparatus, whereas low *PtTOC* during winter permits full deployment of the cold-acclimation machinery while deferring reproductive development. The obverse side of this trade-off was revealed by thermotolerance assays, in which *PtTOC-OE* plants and *PtTOC*-expressing yeast both displayed enhanced growth under heat stress at 37–40 °C, suggesting that *PtTOC* may facilitate adaptation to high-temperature environments through currently uncharacterized mechanisms — a finding that acquires heightened relevance under projected scenarios of increasing thermal extremes associated with climate change. Understanding the regulatory nodes through which *PtTOC* modulates the balance between reproductive readiness and environmental resilience will therefore be of both fundamental biological significance and practical value for developing climate-adapted conifer breeding strategies.

A final, unanticipated finding of the present study concerns the subcellular behavior of *PtTOC*. Confocal imaging of GFP-tagged *PtTOC* in *N. benthamiana* nuclei revealed dynamic morphological transitions — including the formation of discrete, mobile nuclear foci — consistent with liquid-liquid phase separation (LLPS), a biophysical process in which proteins bearing intrinsically disordered regions (IDRs) or multivalent interaction domains condense into membrane-less compartments that concentrate biochemical activities and confer regulatory specificity [60–62]. Phase separation has emerged as a fundamental organizing principle in transcriptional regulation, with condensate formation governing enhancer–promoter communication, co-activator recruitment, and the spatiotemporal gating of gene expression programs. Although LLPS has not been previously reported for plant circadian clock proteins, the phosphorylation of *TOC1* has been shown in *A. thaliana* to be essential for both its clock function and its interactions with the *NF-YB/C* complex [14], and it is well-established that phosphorylation can either promote or dissolve protein condensates depending on the precise modification site and the surrounding molecular context [62]. Prediction results from the NetPhos-3.1 online platform also indicate an abundance of phosphorylation sites within *PtTOC* (Figure S5 and Table S8). Thus, it is likely that *PtTOC* in *P. tabuliformis* also undergoes phosphorylation, regulating circadian rhythms, flowering time, and other vital processes through phase separation in response to environmental cues. These findings represent significant discovery that warrants further investigation for a deeper understanding of these processes and could have practical applications in breeding conifers with improved resilience to environmental stresses.

## Conclusion

In this study, we performed a genome-wide identification of the RR gene family in *P. tabuliformis*, uncovering 35 members classified into 17 A-ARRs, 16 B-ARRs, and 2 pseudo-RRs. Phylogenetic, structural, and expression analyses revealed that the pseudo-RR subfamily is highly conserved in conifers, with only 2 retained members—markedly fewer than in angiosperms. In contrast, A- and B-ARRs have undergone gene expansion and structural diversification, suggesting enhanced functional versatility and highlighting a distinct evolutionary path for RR genes in gymnosperms.

**Fig. 10:**
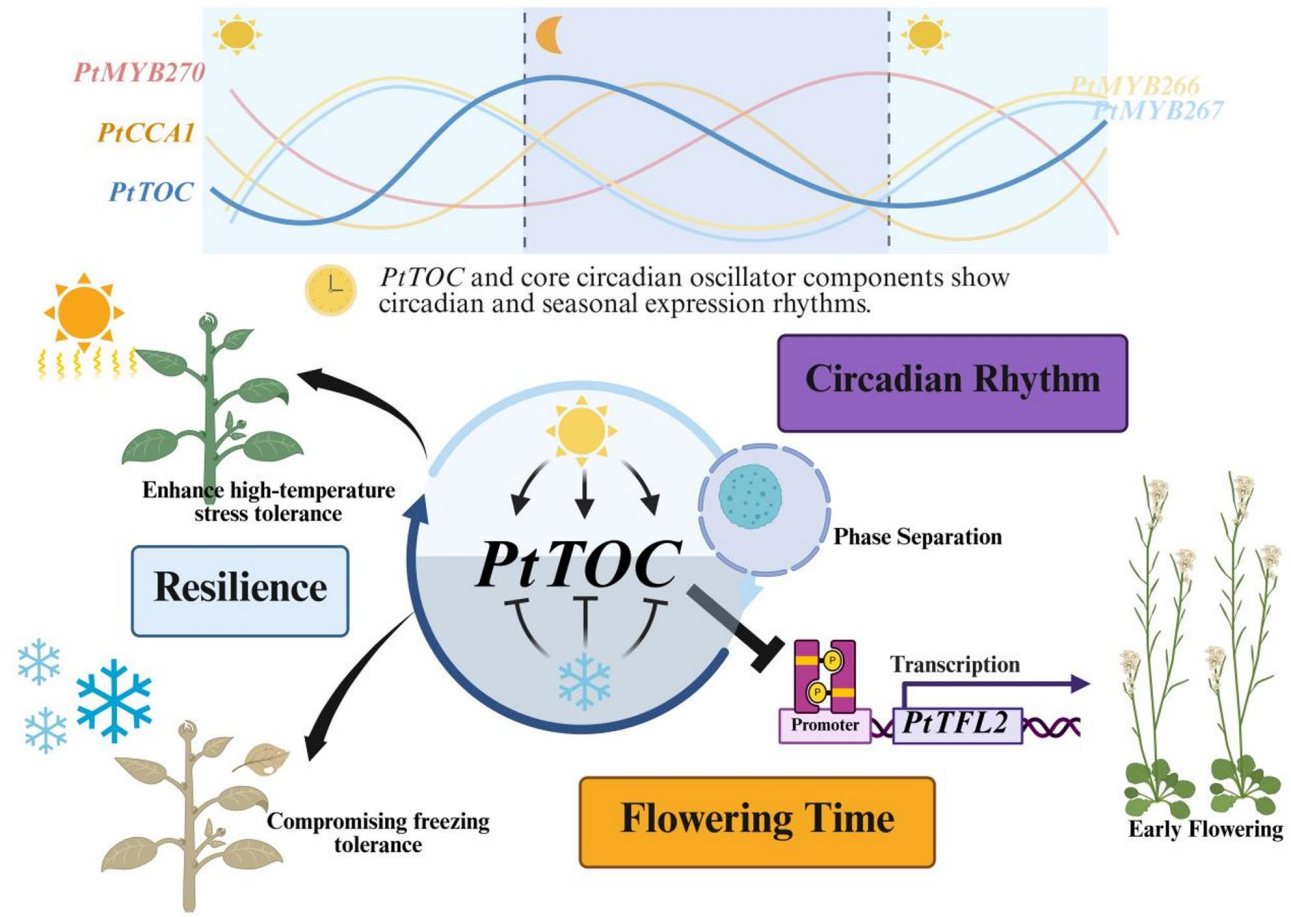
A hypothetical model for PtTOC function in flowering and stress adaptation

Building on this genomic framework, we further investigated the core circadian regulator *PtTOC* and established a model for its role in coordinating environmental signals to regulate flowering (Fig. 10). *PtTOC* and circadian clock homolog genes (*PtCCA1*, *PtMYB266*, *PtMYB267*, *PtMYB270*) exhibit strong diurnal and seasonal expression rhythms. *PtTOC* expression is shaped by photoperiod and temperature: long days promote *PtTOC* accumulation, whereas low temperatures suppress it. Functionally, *PtTOC* acts as a transcriptional repressor that directly binds to the promoter of the floral repressor *PtTFL2*, inhibiting its expression and thereby promoting floral transition. Notably, *PtTOC* overexpression enhanced thermotolerance but compromised freezing tolerance, indicating a trade-off between flowering regulation and stress adaptation. 

In summary, we define the unique evolutionary trajectory of the RR family in gymnosperms and uncover *PtTOC*’s role as a key regulatory node. This work thus provides fundamental insights into the molecular mechanisms controlling conifer flowering and adaptation. 

## Supplementary Information

Supplementary Material 1. Figure. S1. Expression profiles of 19 RR genes exhibiting generally low expression levels in various organs of P. tabuliformis. The analyzed samples included: hypocotyls (n = 36), seedling needles (n = 90), sapling needles (n = 165), Sapling shoot apex (n = 51), Sapling stem cambium (n = 51), Adult needle(n = 468), Adult shoot apex (n = 69), Adult root (n = 16), Adult stem cambium (n = 51), Adult branch cambium (n = 60), Adult vegetative bud (n = 21), Male cone (n = 60), Female cone (n = 18), Embryo (n = 6), Pollen (n = 3), Ovule (n = 24), Callus (n = 18).

Supplementary Material 2. Figure. S2. LUC/REN value represents promoter activity (data are shown as mean ± SD (n = 3). Statistical significance was determined using Student's t-test (*p < 0.05, **p < 0.01).

Supplementary Material 3. Figure. S3. Annual cycle expression profiles of PtCCA1 and PtLHY-like genes. (This figure presents the annual expression profiles of PtCCA1 and the PtLHY-like genes (PtMYB266, PtMYB267, and PtMYB270) in the needles of P. tabuliformis. The monitoring period extended over two years, from July 1, 2017, to July 1, 2019. Error bars indicate the variability observed across three independent replicates).

Supplementary Material 4. Figure. S4. The expression of PtCCA1, PtMYB266, PtMYB267 and PtMYB270 at 8:00, 12:00, 16:00, 20:00, 24:00 and 4:00 in March 25, June 25, July 25, August 25, September 25 and December 25. Error bars represented variability of three independent replicates.

Supplementary Material 5. Figure. S5. PtTOC protein phosphorylation site prediction.

Supplementary Material 6.

Supplementary Material 7.

Supplementary Material 8.

Supplementary Material 9.

Supplementary Material 10.

Supplementary Material 11.

Supplementary Material 12.

Supplementary Material 13.

## Data Availability

The data presented in the study are deposited in the NCBI repository GenBank (accession numbers PRJNA1170861 and PRJNA1171127). Any additional information required to reanalyze the data reported in this work paper is available from the Wei Li and Shihui Niu upon request.

## References

[1] Kakimoto T. Perception and signal transduction of cytokinins. Annu Rev Plant Biol. 2023;54(1):605–27. 10.1146/annurev.arplant.54.031902.134802. 10.1146/annurev.arplant.54.031902.134802 14503005

[2] Zhao JZ, Wang JQ, Li J, Zhang PH, Kudoyarova GZ, Liu CJ, Zhang KW. Spatially distributed cytokinins: metabolism signaling, and transport. Plant Commun. 2024;5(7):100936. 10.1016/j.xplc.2024.100936. 38689499 10.1016/j.xplc.2024.100936 PMC11287186

[3] Acheampong AK, Shanks C, Cheng CY, Schaller GE, Dagdas Y, Kieber JJ. EXO70D isoforms mediate selective autophagic degradation of type-A ARR proteins to regulate cytokinin sensitivity. PNAS. 2020;117(43):27034–43. 10.1073/pnas.2013161117. 33051300 10.1073/pnas.2013161117 PMC7604425

[4] Huang X, Hou L, Meng J, You H, Li Z, Gong Z, Yang S, Shi Y. The antagonistic action of abscisic acid and cytokinin signaling mediates drought stress response in *Arabidopsis*. Mol Plant. 2018;11(7):970–82. 10.1016/j.molp.2018.05.001. 29753021 10.1016/j.molp.2018.05.001

[5] Wang Y, Jiao Y. Auxin and above-ground meristems. J EXP BOT. 2018;69(2):147–54. 10.1093/jxb/erx299. 28992121 10.1093/jxb/erx299

[6] Chen D, Hou L, Qiu Z, Xu Q, Wang Q, Li M, Hao Z, et al. Prime editing to improve the expression of *DAHPS2* in the shikimate pathway by the type-B cytokinin response regulator *RR26* enhances submergence tolerance in rice. Plant Commun. 2026;101714. 10.1016/j.xplc.2026.101714. 10.1016/j.xplc.2026.101714 PMC13084075 41517870

[7] Millar AJ, Carre IA, Strayer CA, Chua NH, Kay SA. Circadian clock mutants in *Arabidopsis* identified by luciferase imaging. Science. 1995;267(5201):1161–3. 10.1126/science.7855595. 7855595 10.1126/science.7855595

[8] Matsushika A, Makino S, Kojima M, Yamashino T, Mizuno T. The APRR1/TOC1 quintet implicated in circadian rhythms of Arabidopsis thaliana: II. Characterization with CCA1-overexpressing plants. Plant Cell Physiol. 2002;43(1):118–22. 10.1093/pcp/pcf006. 11828029 10.1093/pcp/pcf006

[9] Steed G, Ramirez DC, Hannah MA, Webb AR. Chronoculture, harnessing the circadian clock to improve crop yield and sustainability. Science. 2021;372(6541):479. 10.1126/science.abc9141. 10.1126/science.abc9141 33926926

[10] Li J, Qiu JX, Zeng QH, Zhuang Y, Zhang N, Xu SX, Jin J, Dong ZC, Chen L, Huang W. *OsTOC1* plays dual roles in the regulation of plant circadian clock by functioning as a direct transcription activator or repressor. Cell Rep. 2023;42(7):112765. 10.1016/j.celrep.2023.112765. 37421622 10.1016/j.celrep.2023.112765

[11] Wang F, Han T, Chen ZJ. Circadian and photoperiodic regulation of the vegetative to reproductive transition in plants. Commun Bilo. 2024;7(1):1–11. 10.1038/s42003-024-06275-6. 10.1038/s42003-024-06275-6 PMC11098820 38755402

[12] Gauley A, Pasquariello M, Yoshikawa GV, Alabdullah AK, Hayta S, Smedley MA, et al. Photoperiod-1 regulates the wheat inflorescence transcriptome to influence spikelet architecture and flowering time. Curr Biol. 2024;34(11):2330–43. 10.1016/j.cub.2024.04.029. 38781956 10.1016/j.cub.2024.04.029 PMC11149547

[13] Gendron JM, Pruneda-Paz JL, Doherty CJ, Gross AM, Kang SE, Kay SA. *Arabidopsis* circadian clock protein, *TOC1*, is a DNA-binding transcription factor. PNAS. 2012;109(8):3167–72. 10.1073/pnas.1200355109. 22315425 10.1073/pnas.1200355109 PMC3286946

[14] Yan JP, Li SB, Kim YJ, Zeng QN, Radziejwoski A, Wang L, Nomura Y, Nakagami H, Somers DE. *TOC1* clock protein phosphorylation controls complex formation with *NF-YB/C* to repress hypocotyl growth. Embo J. 2021;40(24):23. 10.15252/embj.2021108684. 10.15252/embj.2021108684 PMC8672182 34726281

[15] Yang MK, Lin WJ, Xu YR, Xie BY, Yu BY, Chen L, Huang W. Flowering-time regulation by the circadian clock: from *Arabidopsis* to crops. Crop J. 2024;12(1):17–27. 10.1016/j.cj.2023.09.002.

[16] Liu L, Xuan L, Jiang Y, Yu H. Regulation by flowering locus t and termina*l flower1* in flowering time and plant architecture. 2021;2(4):2000125. 10.1002/sstr.202000125

[17] Sawa M, Kay SA. *GIGANTEA* directly activates *Flowering Locus T* in *Arabidopsis thaliana*. PNAS. 2021;108(28):11698–703. 10.1073/pnas.1106771108. 10.1073/pnas.1106771108 PMC3136272 21709243

[18] Zhang LL, Luo A, Davis SJ, Liu JX. Timing to grow: roles of clock in thermomorphogenesis. Trends Plant Sci. 2021;26(12):1248–57. 10.1016/j.tplants.2021.07.020. 34404586 10.1016/j.tplants.2021.07.020

[19] Kim H, Kang DY, Hwang HW, Lee N, Kubota A, Imaizumi T, Song YH. Low temperature-mediated repression and far-red light-mediated induction determine morning *Flowering locus T* expression levels. J Integr Plant Biol. 2024;66(1):103–20. 10.1111/jipb.13595. 38088490 10.1111/jipb.13595 PMC10829767

[20] Zhu Y, Klasfeld S, Jeong CW, Jin R, Goto K, Yamaguchi N, Wagner D. *Terminal flower 1*-*FD* complex target genes and competition with *flowering locus t*. Nat. Commun. 2020;11:5118. 10.1038/s41467-020-18782-1. 10.1038/s41467-020-18782-1 PMC7550357 33046692

[21] Klintenäs M, Pin P, Benlloch R, Ingvarsson PK, Nilsson O. Analysis of conifer *flowering locus t/terminal flower1-like* genes provides evidence for dramatic biochemical evolution in the angiosperm *FT* lineage. 2012;196(4):1260–1273. 10.1111/j.1469-8137.2012.04332.x 10.1111/j.1469-8137.2012.04332.x 23020222

[22] Niu SH, Yuan HW, Sun X, Porth I, Li Y, El-Kassaby YA, Li W. A transcriptomics investigation into pine reproductive organ development. New Phytol. 2016;209(3):1278–89. 10.1111/nph.13680. 26406997 10.1111/nph.13680

[23] Ramirez-Carvajal GA, Morse AM, Davis JM. Transcript profiles of the cytokinin response regulator gene family in Populus imply diverse roles in plant development. New Phytol. 2008;177(1):77–89. 10.1111/j.1469-8137.2007.02240.x. 17944821 10.1111/j.1469-8137.2007.02240.x

[24] Chu ZX, Ma Q, Lin YX, Tang XL, Zhou YQ, Zhu SW, Fan J, Cheng BJ. Genome-wide identification, classification, and analysis of two-component signal system genes in maize. Genet Mol Res. 2011;10(4):3316–30. 10.3389/fpls.2022.1091620. 22194197 10.4238/2011.December.8.3

[25] Chen C, Liu AL, Re H, Yu Y, Duanmu HZ, Duan XB, Sun XL, Liu BD, Zhu YM. Genome-wide analysis of *Glycine soja* Response Regulator GsRR genes under alkali and salt stresses. Front. Plant Sci. 2018;9:1306. 10.3389/fpls.2018.01306. 10.3389/fpls.2018.01306 PMC6137175 30245700

[26] Lv J, Dai CB, Wang WF, Sun YH. Genome-wide identification of the *ARRs* gene family in tobacco (*Nicotiana tabacum*). Genes Genom. 2021;43(6):601–12. 10.1007/s13258-021-01039-6. 10.1007/s13258-021-01039-6 33772744

[27] Álvarez JM, Cortizo M, Ordás RJ. Characterization of a type-A response regulator differentially expressed during adventitious caulogenesis in *Pinus pinaster*. J plant physiol. 2012;169(18):1807–14. 10.1016/j.jplph.2012.07.014. 22959674 10.1016/j.jplph.2012.07.014

[28] Zhou C, Sun F, Jiao Z, El-Kassaby YA, Li W. Design strategy of advanced generation breeding population of *Pinus tabuliformis* based on genetic variation and inbreeding level. For Ecosyst. 2025;13:100320. 10.1016/j.fecs.2025.100320.

[29] Niu SH, Li J, Bo WH, Yang WF, Zuccolo A, et al. The Chinese pine genome and methylome unveil key features of conifer evolution. Cell. 2022;185(1):204–17. 10.1016/j.cell.2021.12.006. 34965378 10.1016/j.cell.2021.12.006

[30] Chen C, Wu Y, Li J, Zeng X, Xu ZH, Liu J, Feng YL, Chen JTH, He YH, Xia R. TBtools-II: a one for all, all for onebioinformatics platform for biological big-data mining. Mol Plant. 2023;16(11):1733–42. 10.1016/j.molp.2023.09.010. 37740491 10.1016/j.molp.2023.09.010

[31] Abramson J, Adler J, Dunger J, Evans R, Green T, Pritzel A, Ronneberger O, et al. Accurate structure prediction of biomolecular interactions with AlphaFold 3. Nature. 2024;630(8016):493–500. 10.1038/s41586-024-07487-w. 38718835 10.1038/s41586-024-07487-w PMC11168924

[32] Liu HM, Guo YT, Wang HL, Yang WB, Yang JH, Zhang JX, Liu D, El-Kassaby YA, Li W. Involvement of *PtCOL5-PtNF-YC4* in reproductive cone development and gibberellin signaling in Chinese pine. Plant Sci. 2022;323:111383. 10.1016/j.plantsci.2022.111383. 35850285 10.1016/j.plantsci.2022.111383

[33] Jiao Z, Tian Y, Cao Y, Wang J, Zhan B, Zhao Z, Sun B, Guo C, Ma W, Liao Z, Zhang H, Zhou T, Xia Y, Fan Z. A novel pathogenicity determinant hijacks maize catalase 1 to enhance viral multiplication and infection. New Phytol. 2021;230(3):1126–41. 10.1111/nph.17206. 33458828 10.1111/nph.17206

[34] Zhou C, Liu H, Wang H, Niu S, El-Kassaby YA, Li W. Deciphering the role of *SVP-Like* genes and their key regulation networks during reproductive cone development in *Pinus tabuliformis*. Plant cell environ. 2024;48(1):365–86. 10.1111/pce.15129. 39257299 10.1111/pce.15129

[35] Li C, He YQ, Yu J, Kong JR, Ruan CC, Yang ZK, Zhuang JJ, Wang YX, Xu JH. The rice *late elongated hypocotyl* enhances salt tolerance by regulating Na+/K+ homeostasis and ABA signalling. Plant cell environ. 2024;47(5):1625–39. 10.1111/pce.14835. 38282386 10.1111/pce.14835

[36] Zheng Q, Yu Q, Yao W, Lv K, Zhang N, Xu W. Decoding *VaCOLD1* Function in grapevines: a membrane protein enhancing cold stress tolerance. J Agric Food Chem. 2023;71(49):19357–71. 10.1021/acs.jafc.3c05101. 38037352 10.1021/acs.jafc.3c05101

[37] Qiang YQ, He XJ, Li Z, Li SQ, Zhang J, Liu T, Tursunniyaz M, Wang XY, Liu ZP, Fang LF. Genome-wide identification and expression analysis of the response regulator gene family in alfalfa (*Medicago sativa* L.) reveals their multifarious roles in stress response. Front Plant Sci. 2023;14:1149880. 10.3389/fpls.2023.1149880. 36998691 10.3389/fpls.2023.1149880 PMC10043395

[38] Shigeru H, Koji G. Arabidopsis *terminal flower1* is involved in the regulation of flowering time and inflorescence development through transcriptional repression. Plant Cell. 2011;23(9):3172–3184. 10.1105/tpc.111.088641 10.1105/tpc.111.088641 PMC3203435 21890645

[39] Qu K, Zhou C, Liu D, Han B, Jiao Z, Niu S, El-Kassaby YA, Li W. *CONSTANS-Like* and short vegetative phase‐like genes coordinately modulate *terminal flower 2* to control dormancy transitions in *Pinus tabuliformis*. Plant Cell Environ. 2024;48(5):3066–84. 10.1111/pce.15313. 39676713 10.1111/pce.15313

[40] Yang J, Qu K, Wang HL, El-Kassaby YA, Li W. Diurnal dynamics of different circadian transcription modules in Chinese pine needles and roots during dormancy induction. BMC Plant Biol. 2025;25(1):413. 10.1186/s12870-025-06365-5. 40170165 10.1186/s12870-025-06365-5 PMC11963403

[41] Jang J, Lee S, Kim J-I, Lee S, Kim JA. The roles of circadian clock genes in plant temperature stress responses. Int J Mol Sci. 2024;25(2):918. 10.3390/ijms25020918. 38255990 10.3390/ijms25020918 PMC10815334

[42] Zhou C, Liu H, Sang Y, Jiang HR, Zhang JS, Niu SH, El-Kassaby YA, Li W. Short-day-induced expression of *PtTFL2* triggers growth-defense tradeoffs during *Pinus tabuliformis* dormancy. Plant Physiol. 2025;198(4):kiaf362. 10.1093/plphys/kiaf362. 40826501 10.1093/plphys/kiaf362

[43] Hwang I, Sheen J, Mueller B. Cytokinin signaling networks. Ann Rev Plant Biol. 2012;63:353–80. 10.1146/annurev-arplant-042811-105503. 22554243 10.1146/annurev-arplant-042811-105503

[44] Tuskan GA, DiFazio S, Jansson S, Bohlmann J, Grigoriev I, et al. The genome of black cottonwood, *Populus trichocarpa*. Science. 2006;313(5793):1596–604. 10.1126/science.1128691. 16973872 10.1126/science.1128691

[45] Frohlich MW, Chase MW. After a dozen years of progress the origin of angiosperms is still a great mystery. Nature. 2007;450(7173):1184–9. 10.1038/nature06393. 18097399 10.1038/nature06393

[46] Bateman RM, Hilton J, Rudall PJ. Spatial separation and developmental divergence of male and female reproductive units in gymnosperms, and their relevance to the origin of the angiosperm flower. Flowers Tree Life. 2011;41:8–48. 10.1017/CBO9781139013321.002.

[47] Scutt CP, Vandenbussche M. Current trends and future directions in flower development research. Ann Bot. 2014;114(7):1399–406. 10.1093/aob/mcu224. 25335868 10.1093/aob/mcu224 PMC4204790

[48] Shen L, Yang C, Li D, An M, Xiao F, Cai M, et al. Genome-wide analysis of the type-B ARRs gene family reveals key regulators of abiotic stress tolerance and hormone signaling in *Medicago truncatula*. Int J Biol Macromol. 2026;340(2):150026. 10.1016/j.ijbiomac.2025.150026. 41520972 10.1016/j.ijbiomac.2025.150026

[49] Mähönen MH, Miyawaki K, Hashimoto Y, Seki M, Kobayashi M, Shinozaki K, Kato T, Tabata S, Helariutta Y, Sussman MR, Kakimoto T. In planta functions of the *Arabidopsis* cytokinin receptor family. PNAS. 2004;101(23):8821–6. 10.1073/pnas.0402887101. 15166290 10.1073/pnas.0402887101 PMC423279

[50] Argyros RD, Mathews DE, Chiang YH, Palmer CM, Thibault DM, Etheridge N, Argyros DA, Mason MG, Kieber JJ, Schaller GE. Type B response regulators of arabidopsis play key roles in cytokinin signaling and plant development. Plant Cell. 2008;20(8):2102–16. 10.1105/tpc.108.059584. 18723577 10.1105/tpc.108.059584 PMC2553617

[51] Tu TL, Zheng SS, Ren PR, Meng XW, Zhao JH, Chen Q, Li CY. Coordinated cytokinin signaling and auxin biosynthesis mediates arsenate-induced root growth inhibition. Plant Physiol. 2021;185(3):1166–81. 10.1093/plphys/kiaa072. 33793921 10.1093/plphys/kiaa072 PMC8133639

[52] Liu ZH, Dai XH, Li J, Liu N, Liu XZ, Li S, Xiang FN. The Type-B cytokinin response regulator *ARR1* inhibits shoot regeneration in an *ARR12*-dependent manner in *Arabidopsis*. Plant Cell. 2020;32(7):2271–91. 10.1105/tpc.19.00022. 32398274 10.1105/tpc.19.00022 PMC7346554

[53] Ma J, Chen X, Han F, Song Y, Zhou B, Nie Y, Li Y, Niu S. The long road to bloom in conifers. Forestry Res. 2022;2(1):157–66. 10.48130/FR-2022-0016. 10.48130/FR-2022-0016 PMC11524297 39525411

[54] Strayer C, Oyama T, Schultz TF, Raman R, Somers DE, Más P, Panda S, Kreps JA, Kay SA. Cloning of the arabidopsis clock gene *TOC1*, an autoregulatory response regulator homolog. Science. 2000;289(5480):768–71. 10.1126/science.289.5480.768. 10926537 10.1126/science.289.5480.768

[55] Yang MJ, Chen S, Lim SL, Yang L, Zhong JY, Chan KC, Zhao ZZ, Wong KB, Wang JQ, Lim BL. A converged ubiquitin-proteasome pathway for the degradation of *TOC* and *TOM* tail-anchored receptors. J Integr Plant Biol. 2024;66(5):1007–23. 10.1111/jipb.13645. 38501483 10.1111/jipb.13645

[56] Chang YY, Bräutigam K, Hüner NPA, Ensminger I. Champions of winter survival: cold acclimation and molecular regulation of cold hardiness in evergreen conifers. New Phytol. 2021;229(2):675–91. 10.1111/nph.16904. 32869329 10.1111/nph.16904

[57] Kidokoro S, Shinozaki K, Yamaguchi-Shinozaki K. Transcriptional regulatory network of plant cold-stress responses. Trends Plant Sci. 2022;27(9):922–35. 10.1016/j.tplants.2022.01.008. 35210165 10.1016/j.tplants.2022.01.008

[58] Liu J, Shi Y, Yang S. Insights into the regulation of *CBF* cold signaling in plants. J Integr Plant Biol. 2018;60(9):780–95. 10.1111/jipb.12706. 29667328 10.1111/jipb.12657

[59] Du SX, Wang LL, Yu WP, Xu SX, Chen L, Huang W. Appropriate induction of *TOC1* ensures optimal *MYB44* expression in ABA signaling and stress response in *Arabidopsis*. Plant cell environ. 2024;47(8):3046–62. 10.1111/pce.14922. 38654596 10.1111/pce.14922

[60] Liu Q, Liu W, Niu Y, Wang T, Dong J. Liquid-liquid phase separation in plants: advances and perspectives from model species to crops. Plant Commun. 2024;5(1):100663. 10.1016/j.xplc.2023.100663. 37496271 10.1016/j.xplc.2023.100663 PMC10811348

[61] Hutin S, Kumita JR, Strotmann VI, Dolata A, Ling WL, Louafi N, et al. Phase separation and molecular ordering of the prion-like domain of the *Arabidopsis* thermosensory protein EARLY FLOWERING 3. PNAS. 2023;120(28):e2304714120. 10.1073/pnas.2304714120. 37399408 10.1073/pnas.2304714120 PMC10334799

[62] Kim TH, Tsang B, Vernon RM, Sonenberg N, Kay LE, Forman-Kay JD. Phospho-dependent phase separation of *FMRP* and *CAPRIN1* recapitulates regulation of translation and deadenylation. Science. 2019;365(6455):825–9. 10.1126/science.aax4240. 31439799 10.1126/science.aax4240

