# Supplementary material for: A circadian repressor promotes flowering via dual repression: PtTOC suppresses the floral inhibitor PtTFL2 in Pinus tabuliformis
Source: BMC Plant Biol. 2026 Mar 19;26:758. doi: 10.1186/s12870-026-08511-z (PMC13123111; doi:10.1186/s12870-026-08511-z)
Supplement: Supplementary file 1 — Supplementary Material 1. Figure. S1. Expression profiles of 19 RR genes exhibiting generally low expression levels in various organs of P. tabuliformis. The analyzed samples included: hypocotyls (n = 36), seedling needles (n = 90), sapling needles (n = 165), Sapling shoot apex (n = 51), Sapling stem cambium (n = 51), Adult needle(n = 468), Adult shoot apex (n = 69), Adult root (n = 16), Adult stem cambium (n = 51), Adult branch cambium (n = 60), Adult vegetative bud (n = 21), Male cone (n = 60), Female cone (n = 18), Embryo (n = 6), Pollen (n = 3), Ovule (n = 24), Callus (n = 18). [file 12870_2026_8511_MOESM1_ESM.zip › Supplementary/Table S1.docx]

**Table S1 Amino acid sequences of members of the RR gene family in *A. thaliana***

**A-ARR**

>AT1G59940.1

MAKDGGVSCLRRSEMIGIGIGELESPPLDSDQVHVLAVDDSLVDRIVIERLLRITSCKVT

AVDSGWRALEFLGLDDDKAAVEFDRLKVDLIITDYCMPGMTGYELLKKIKESTSFKEVPV

VIMSSENVMTRIDRCLEEGAEDFLLKPVKLADVKRLRSYLTRDVKVAAEGNKRKLTTPPP

PPPLSATSSMESSDSTVESPLSMVDDEDSLTMSPESATSLVDSPMRSPGLA

>AT1G10470.1

MARDGGVSCLRRSEMMSVGGIGGIESAPLDLDEVHVLAVDDSLVDRIVIERLLRITSCKV

TAVDSGWRALEFLGLDNEKASAEFDRLKVDLIITDYCMPGMTGYELLKKIKESSNFREVP

VVIMSSENVLTRIDRCLEEGAQDFLLKPVKLADVKRLRSHLTKDVKLSNGNKRKLPEDSS

SVNSSLPPPSPPLTISPESSPPLTVSTESSDSSPPLSPVEIFSTSPLSSPIDDEDDDVLT

SSSEESPIRRQKMRSPGLD

>AT3G48100.1

MAEVLRPEMLDISNDTSSLASPKLLHVLAVDDSMVDRKFIERLLRVSSCKVTVVDSATRA

LQYLGLDGENNSSVGFEDLKINLIMTDYSMPGMTGYELLKKIKESSAFREIPVVIMSSEN

ILPRIDRCLEEGAEDFLLKPVKLADVKRLRDSLMKAEERAFKNIMHKRELEANDIYSQLK

RAKI

>AT5G62920.1

MAEVMLPRKMEILNHSSKFGSPDPLHVLAVDDSHVDRKFIERLLRVSSCKVTVVDSATRA

LQYLGLDVEEKSVGFEDLKVNLIMTDYSMPGMTGYELLKKIKESSAFREVPVVIMSSENI

LPRIDRCLEEGAEDFLLKPVKLSDVKRLRDSLMKVEDLSFTKSIQKRELETENVYPVHSQ

LKRAKI

>AT1G19050.1

MAVGEVMRMEIPAGGDLTVTTPELHVLAVDDSIVDRKVIERLLRISSCKVTTVESGTRAL

QYLGLDGGKGASNLKDLKVNLIVTDYSMPGLSGYDLLKKIKESSAFREVPVVIMSSENIL

PRIQECLKEGAEEFLLKPVKLADVKRIKQLIMRNEAEECKILSHSNKRKLQEDSDTSSSS

HDDTSIKDSSCSKRMKSESENLFSLL

>AT2G41310.1

MVMETESKFHVLAVDDSLFDRKMIERLLQKSSCQVTTVDSGSKALEFLGLRVDDNDPNALSTSPQIHQEVEINLIITDYCMPGMTGYDLLKKVKESAAFRSIPVVIMSSENVPARISRCL

EEGAEEFFLKPVKLADLTKLKPHMMKTKLKKESEKPVAIEEIVVSKPEIEEEEEESSVIE

ILPLHQEIESEQLEPMLSSNKRKAMEEVVSTDRSRPKYNDITTSV

>AT3G57040.1

MGMAAESQFHVLAVDDSLFDRKLIERLLQKSSCQVTTVDSGSKALEFLGLRQSTDSNDPN

AFSKAPVNHQVVEVNLIITDYCMPGMTGYDLLKKVKESSAFRDIPVVIMSSENVPARISR

CLEEGAEEFFLKPVRLADLNKLKPHMMKTKLKNQKLEEIETTSKVENGVPTAVADPEIKD

STNIEIEILPLQQDLLLVQQEEQTLSINNKRKSVEEGISTDRARPRFDGIATAV

>AT1G74890.1

MALRDLSSSLSSSSSPELHVLAVDDSFVDRKVIERLLKISACKVTTVESGTRALQYLGLD

GDNGSSGLKDLKVNLIVTDYSMPGLTGYELLKKIKESSALREIPVVIMSSENIQPRIEQC

MIEGAEEFLLKPVKLADVKRLKELIMRGGEAEEGKTKKLSPKRILQNDIDSSPSSSSTSS

SSSSHDVSSLDDDTPSSKRIKLESRG

>AT2G40670.1

MNSSGGSCSSLMDVVAYDHHLHHGHDEELHVLAVDDNLIDRKLVERLLKISCCKVTTAEN

ALRALEYLGLGDQNQHIDALTCNVMKVSLIITDYCMPGMTGFELLKKVKESSNLREVPVVIMSSENIPTRINKCLASGAQMFMQKPLKLADVEKLKCHLMNCRS

>AT3G56380.1

MNKGCGSGSDSCLSSMEEELHVLAVDDNLIDRKLVERILKISSCKVTTAENGLRALEYLG

LGDPQQTDSLTNVMKVNLIITDYCMPGMTGFELLKKVKESSNLKEVPVVILSSENIPTRI

NKCLASGAQMFMQKPLKLSDVEKLKCHLLNCRS

**B-ARR**

>AT3G16857.1

MMNPSHGRGLGSAGGSSSGRNQGGGGETVVEMFPSGLRVLVVDDDPTCLMILERMLRTCLYEVTKCNRAEMALSLLRKNKHGFDIVISDVHMPDMDGFKLLEHVGLEMDLPVIMMSADDSKSVVLKGVTHGAVDYLIKPVRMEALKNIWQHVVRKRRSEWSVPEHSGSIEETGERQQQQHRGGGGGAAVSGGEDAVDDNSSSVNEGNNWRSSSRKRKDEEGEEQGDDKDEDASNLKKPRVVWSVELHQQFVAAVNQLGVEKAVPKKILELMNVPGLTRENVASHLQKYRIYLRRLGGVSQHQGNLNNSFMTGQDASFGPLSTLNGFDLQALAVTGQLPAQSLAQLQAAGLGRPAMVSKSGLPVSSIVDERSIFSFDNTKTRFGEGLGHHGQQPQQQPQMNLLHGVPTGLQQQLPMGNRMSIQQQIAAVRAGNSVQNNGMLMPLAGQQSLPRGPPPMLTSSQSSIRQPMLSNRISERSGFSGRNNIPESSRVLPTSYTNLTTQHSSSSMPYNNFQPELPVNSFPLASAPGISVPVRKATSYQEEVNSSEAGFTTPSYDMFTTRQNDWDLRNIGIAFDSHQDSESAAFSASEAYSSSSMSRHNTTVAATEHGRNHQQPPSGMVQHHQVYADGNGGSVRVKSERVATDTATMAFHEQYSNQEDLMSALLKQV

>AT4G31920.1

MTMEQEIEVLDQFPVGMRVLAVDDDQTCLRILQTLLQRCQYHVTTTNQAQTALELLRENKNKFDLVISDVDMPDMDGFKLLELVGLEMDLPVIMLSAHSDPKYVMKGVKHGACDYLLKPVRIEELKNIWQHVVRKSKLKKNKSNVSNGSGNCDKANRKRKEQYEEEEEEERGNDNDDPTAQKKPRVLWTHELHNKFLAAVDHLGVERAVPKKILDLMNVDKLTRENVASHLQKFRVALKKVSDDAIQQANRAAIDSHFMQMNSQKGLGGFYHHHRGIPVGSGQFHGGTTMMRHYSSNRNLGRLNSLGAGMFQPVSSSFPRNHNDGGNILQGLPLEELQINNNINRAFPSFTSQQNSPMVAPSNLLLLEGNPQSSSLPSNPGFSPHFEISKRLEHWSNAALSTNIPQSDVHSKPDTLEWNAFCDSASPLVNPNLDTNPASLCRNTGFGSTNAAQTDFFYPLQMNQQPANNSGPVTEAQLFRSSNPNEGLLMGQQKLQSGLMASDAGSLDDIVNSLMTQEQSQSDFSEGDWDLDGLAHSEHAYEKLHFPFSLSA

>AT1G67710.1

MEKSGFSPVGLRVLVVDDDPTWLKILEKMLKKCSYEVTTCGLAREALRLLRERKDGYDIVISDVNMPDMDGFKLLEHVGLELDLPVIMMSVDGETSRVMKGVQHGACDYLLKPIRMKELKIIWQHVLRKKLQEVRDIEGCGYEGGADWITRYDEAHFLGGGEDVSFGKKRKDFDFEKKLLQDESDPSSSSSKKARVVWSFELHHKFVNAVNQIGCDHKAGPKKILDLMNVPWLTRENVASHLQKYRLYLSRLEKGKELKCYSGGVKNADSSPKDVEVNSGYQSPGRSSYVFSGGNSLIQKATEIDPKPLASASLSDLNTDVIMPPKTKKTRIGFDPPISSSAFDSLLPWNDVPEVLESKPVLYENSFLQQQPLPSQSSYVANSAPSLMEEEMKPPYETPAGGSSVNADEFLMPQDKIPTVTLQDLDPSAMKLQEFNTEAILRSLNWELPESHHSVSLDTDLDLTWLQGERFLANTGLQFQDYSSSPSLLSELPAHLNWYGNERLPDPDEYSFMVDQGLFIS

>AT2G25180.1

MTVEQNLEALDQFPVGMRVLAVDDDQTCLKILESLLRHCQYHVTTTNQAQKALELLRENKNKFDLVISDVDMPDMDGFKLLELVGLEMDLPVIMLSAHSDPKYVMKGVTHGACDYLLKPVRIEELKNIWQHVVRSRFDKNRGSNNNGDKRDGSGNEGVGNSDQNNGKGNRKRKDQYNEDEDEDRDDNDDSCAQKKQRVVWTVELHKKFVAAVNQLGYEKAMPKKILDLMNVEKLTRENVASHLQKFRLYLKRISGVANQQAIMANSELHFMQMNGLDGFHHRPIPVGSGQYHGGAPAMRSFPPNGILGRLNTPSGIGVRSLSSPPAGMFLQNQTDIGKFHHVSSLPLNHSDGGNILQGLPMPLEFDQLQTNNNKSRNMNSNKSIAGTSMAFPSFSTQQNSLISAPNNNVVVLEGHPQATPPGFPGHQINKRLEHWSNAVSSSTHPPPPAHNSNSINHQFDVSPLPHSRPDPLEWNNVSSSYSIPFCDSANTLSSPALDTTNPRAFCRNTDFDSNTNVQPGVFYGPSTDAMALLSSSNPKEGFVVGQQKLQSGGFMVADAGSLDDIVNSTMKQEQSQGDLSGGDLGYGGFSSLRTCI

>AT2G27070.1

MAFAQSVYNQSSVLKINVMVVDDNRVFLDIWSRMLEKSKYREITVIAVDYPKKALSTLKN

QRDNIDLIITDYYMPGMNGLQLKKQITQEFGNLSVLVMSSDPNKEEESLSCGAMGFIPKP

IAPTDLPKIYQFALTYKRNGKSTLSTEQNQKDANVSVPQQIMLVPEQAYVLKTKKKNCSS

KSDTRTVNSTNVSHVSTNGSRKNRKRKPKGGPSDDGESLSQPPKKKKIWWTNPLQDLFLQAIQHIGYDKVVPKKILAIMNVPYLTRENVASHLQKYRLFVKRVVHQGRFSMLSDRGKDSMFRQTHIKEPYVNYYTPSTSWYETSLNNRSFYSESVHGHSRLLSEAREPVRYNQMSYNYMNRNISFENQPSQNEETRTVFEPPVMANKISQTSQVLGFGQLGPSAISGHNFNTNMMSSYGSLTPNQPGTSHFSYGMQSVLNNENATYNPQPPANATTQPNLDELPQLENLNLYNDLGNTSELPYNISNFQSDDNKKQGEEDGDWTFVNINQDQSNGESSNTIATPETNTPNFNINPNQNQGQAVPEFTDWSFLDQQELVDDDFMNSLFNNDMN

>AT2G01760.1

MPINDQFPSGLRILVVDDDTSCLFILEKMLLRLMYQVTICSQADVALTILRERKDSFDLV

LSDVHMPGMNGYNLLQQVGLLEMDLPVIMMSVDGRTTTVMTGINHGACDYLIKPIRPEELKNIWQHVVRRKCVMKKELRSSQALEDNKNSGSLETVVVSVSECSEESLMKCRNKKKKKKRSVDRDDNEDDLLLDPGNSKKSRVVWSIELHQQFVNAVNKLGIDKAVPKRILELMNVPGLSRENVASHLQKFRLYLKRLSGEASQSNDSESTKRYENIQALVSSGQLHPQTLAALFGQPIDNHHSASFGVWIPNDNLGRSQNEHFSVDVSSASNRPVSVAVHGLSSSANFRQRGDVNNNRIRQGYGSNVNEESWILERSSRQR

>AT5G58080.1

MEFGSTEDGRHDKFPVGMRVLAVDDNPTCLRKLEELLLRCKYHVTKTMESRKALEMLRENSNMFDLVISDVEMPDTDGFKLLEIGLEMDLPVIMLSAHSDYDSVMKGIIHGACDYLVKPVGLKELQNIWHHVVKKNIKSYAKLLPPSESDSVPSASRKRKDKVNDSGDEDDSDREEDDGEGSEQDGDGSGTRKKPRVVWSQELHQKFVSAVQQLGLDKAVPKKILDLMSIEGLTRENVASHLQKYRLYLKKIDEGQQQNMTPDAFGTRDSSYFQMAQLDGLRDFTAARQIPSSGLLSRSHLTKLQPPMYSSINLQGMNSSSFIQQGHHQNSSNSANPFGTYHSTLSPRIQNVNLFQRTSS

PLEPLQFPRSKSYIGDFKGLGDRAIGGSFLDTCMPFGSSSTSLPSASTNPLMLQANYTQP

LHIASDGIQPCIEGTPSNSASPNISFQGLSRFPGHSWQGNLNTTRFPPSSLPLNLAFLPD

QVTCAGNNLGDCTSLVSAENPGGEMQCDPQLLGGFMQNVNPLGGQKWEQQNCTMLNNPFGNIEYPLPADNMVFRDNNSTRSKGLDESLMNPIDNSQEYVGKATTMLDPEMKSGKPENDNQHDVFDDIMNEMMKQEENNGMVPVATRFGFDSFPPP

>AT1G49190.1

MLVGKISGYEDNTRSLERETSEITSLLSQFPGNTNVLVVDTNFTTLLNMKQIMKQYAYQV

SIETDAEKALAFLTSCKHEINIVIWDFHMPGIDGLQALKSITSKLDLPVVIMSDDNQTES

VMKATFYGACDYVVKPVKEEVMANIWQHIVRKRLIFKPDVAPPKPRMTWSEVFQPVQSHLVPTDGLDRDHFDSITINGGNGIQNMEKKQGKKPRKPRMTWTEELHQKFLEAIEIIGGIEK

ANPKVLVECLQEMRIEGITRSNVASHLQKHRINLEENQIPQQTQGNGWATAYGTLAPSLQ

GSDNVNTTIPSYLMNGPATLNQIQQNQYQNGFLTMNNNQIITNPPPPLPYLDHHHQQQHQ

SSPQFNYLMNNEELLQASGLSATDLELTYPSLPYDPQEYLINGYNYN

>AT3G62670.1

MSVFSNILDENSRNLRNEIPCDDGIASPINDDDEEFLTKSNRVLLVGADSNSSLKNLMTQ

YSYQVTKYESGEEAMAFLMKNKHEIDLVIWDFHMPDINGLDALNIIGKQMDLPVVIMSHEYKKETVMESIKYGACDFLVKPVSKEVIAVLWRHVYRKRMSKSGLDKPGESGTVESDPDEYDDLEQDNLYESNEEGSKNTCDHKEEKSPTKKPRMQWTPELHHKFEVAVEKMGSLEKAFPKTILKYMQEELNVQGLTRNNVASHLQKYRQSSKKTCTPQEPQEDFVWGNAGPDVTLAASKTLLSSHATPSYLINNQAAPRGSYFMNNIPYPSTSCLPVNNNNCFMTNPSTYIDQFQHQLQQQQQHQQYQSTLNSISAMLTKQESRHVPSSAMENSEPLMIYNSNLPFGIDECFPPAGFNIFDQIGHN

>AT5G07210.1

MASAQSFYNQSSVLKINVMVVDDDHVFLDIMSRMLQHSKYRDPSVMEIAVIAVDDPKKALSTLKIQRDNIDLIITDYYMPGMNGLQLKKQITQEFGNLPVLVMSSDTNKEEESLSCGAMG

FIPKPIHPTDLTKIYQFALSNKRNGKSTLSTEQNHKDADVSVPQQITLVPEQADVLKTKR

KNCSFKSDSRTVNSTNGSCVSTDGSRKNRKRKPNGGPSDDGESMSQPAKKKKIQWTDSLHDLFLQAIRHIGLDKAVPKKILAFMSVPYLTRENVASHLQKYRIFLRRVAEQGLYSMLSDR

GIDSMFRQTHIKEPYFNYYTPSTSWYDTRLNNRSFYSKPVHGFGQSKLLSTTREPVCFNQ

MPYNYMNRSSTYEPHRIGSGSNLTLPIQSNLSFPNQPSQNEERRSFFEPPVMANKIAQTS

QVLGFGQLGPSAISGHNFNNNMTSRYGSLIPSQPGPSHFSYGMQSFLNNENVTYNPQPPA

NATTQPNLDELPQLENLNLYNDFGNTSELPYNISNFQFDDNKHQQGEADPTKFELPAAKF

STELNHEDDGDWTFVNINQGQSNGETSNTIASPETNTPILNINHNQNQGQDVPEFNDWSF

LDPQELVDDDFMNSLFNNDMN

>AT4G16110.1

MVNPGHGRGPDSGTAAGGSNSDPFPANLRVLVVDDDPTCLMILERMLMTCLYRVTKCNRAESALSLLRKNKNGFDIVISDVHMPDMDGFKLLEHVGLEMDLPVIMMSADDSKSVVLKGVTHGAVDYLIKPVRIEALKNIWQHVVRKKRNEWNVSEHSGGSIEDTGGDRDRQQQHREDADNNSSSVNEGNGRSSRKRKEEEVDDQGDDKEDSSSLKKPRVVWSVELHQQFVAAVNQLGVDKAVPKKILEMMNVPGXTRENVASHLQKYRIYLRRLGGVSQHQGNMNHSFMTGQDQSFGPLSSLNGFDLQSLAVTGQLPPQSLAQLQAAGLGRPTLAKPGMSVSPLVDQRSIFNFENPKIRFGDGHGQTMNNGNLLHGVPTGSHMRLRPGQNVQSSGMMLPVADQLPRGGPSMLPSLGQQPILSSSVSRRSDLTGALAVRNSIPETNSRVLPTTHSVFNNFPADLPRSSFPLASAPGISVPVSVSYQEEVNSSDAKGGSSAATAGFGNPSYDIFNDFPQHQQHNKNISNKLNDWDLRNMGLVFSSNQDAATATATAAFSTSEAYSSSSTQRKRRETDATVVGEHGQNLQSPSRNLYHLNHVFMDGGSVRVKSERVAETVTCPPANTLFHEQYNQEDLMSAFLKQEGIPSVDNEFEFDGYSIDNIQV

**Pseudo-RR**

>AT5G61380.1

MDLNGECKGGDGFIDRSRVRILLCDNDSTSLGEVFTLLSECSYQVTAVKSARQVIDALNA

EGPDIDIILAEIDLPMAKGMKMLRYITRDKDLRRIPVIMMSRQDEVPVVVKCLKLGAADY

LVKPLRTNELLNLWTHMWRRRRMLGLAEKNMLSYDFDLVGSDQSDPNTNSTNLFSDDTDDRSLRSTNPQRGNLSHQENEWSVATAPVHARDGGLGADGTATSSLAVTAIEPPLDHLAGSHHEPMKRNSNPAQFSSAPKKSRLKIGESSAFFTYVKSTVLRTNGQDPPLVDGNGSLHLHRGLAEKFQVVASEGINNTKQARRATPKSTVLRTNGQDPPLVNGNGSHHLHRGAAEKFQVVASEGINNTKQAHRSRGTEQYHSQGETLQNGASYPHSLERSRTLPTSMESHGRNYQEGNMNIPQVAMNRSKDSSQVDGSGFSAPNAYPYYMHGVMNQVMMQSAAMMPQYGHQIPHCQPNHPNGMTGYPYYHHPMNTSLQHSQMSLQNGQMSMVHHSWSPAGNPPSNEVRVNKLDRREEALLKFRRKRNQRCFDKKIRYVNRKRLAERRPRVKGQFVRKMNGVNVDLNGQPDSADYDDEEEEEEEEEEENRDSSPQDDALGT

>AT5G60100.1

MCFNNIETGDEVETERQVFGSSEEDEFRVEDTARNTNNVQISQQQQQPLAHVVKWERYLP

VRSLKVLLVENDDSTRHIVTALLKNCSYEVTAVPDVLEAWRILEDEKSCIDLVLTEVDMP

VHSGTGLLSKIMSHKTLKNIPVIMMSSHDSMVLVFKCLSNGAVDFLVKPIRKNELKNLWQ

HVWRRCHSSSGSGSESGIHDKKSVKPESTQGSENDASISDEHRNESGSSGGLSNQDGGSD

NGSGTQSSWTKRASDTKSTSPSNQFPDAPNKKGTYENGCAHVNRLKEAEDQKEQIGTGSQTGMSMSKKAEEPGDLEKNAKYSVQALERNNDDTLNRSSGNSQVESKAPSSNREDLQSLEQTLKKTREDRDYKVGDRSVLRHSNLSAFSKYNNGATSAKKAPEENVESCSPHDSPIAKLLGSSSSSDNPLKQQSSGSDRWAQREAALMKFRLKRKERCFEKKVRYHSRKKLAEQRPHVKGQFIRKRDDHKSGSEDN

>AT5G24470.1

MTSSEEVVEVTVVKAPEAGGGKLSRRKIRKKDAGVDGLVKWERFLPKIALRVLLVEADDSTRQIIAALLRKCSYRVAAVPDGLKAWEMLKGKPESVDLILTEVDLPSISGYALLTLIMEH

DICKNIPVIMMSTQDSVNTVYKCMLKGAADYLVKPLRRNELRNLWQHVWRRQTSLAPDSFPWNESVGQQKAEGASANNSNGKRDDHVVSGNGGDAQSSCTRPEMEGESADVEVSARDAVQMECAKSQFNETRLLANELQSKQAEAIDFMGASFRRTGRRNREESVAQYESRIELDLSLRRPNASENQSSGDRPSLHPSSASAFTRYVHRPLQTQCSASPVVTDQRKNVAASQDDNIVLMNQYNTSEPPPNAPRRNDTSFYTGADSPGPPFSNQLNSWPGQSSYPTPTPINNIQFRDPNTAYTSAMAPASLSPSPSSVSPHEYSSMFHPFNSKPEGLQDRDCSMDVDERRYVSSATEHSAI

GNHIDQLIEKKNEDGYSLSVGKIQQSLQREAALTKFRMKRKDRCYEKKVRYESRKKLAEQRPRIKGQFVRQVQSTQAP

>AT5G02810.1

MNANEEGEGSRYPITDRKTGETKFDRVESRTEKHSEEEKTNGITMDVRNGSSGGLQIPLS

QQTAATVCWERFLHVRTIRVLLVENDDCTRYIVTALLRNCSYEVVEASNGIQAWKVLEDL

NNHIDIVLTEVIMPYLSGIGLLCKILNHKSRRNIPVIMMSSHDSMGLVFKCLSKGAVDFL

VKPIRKNELKILWQHVWRRCQSSSGSGSESGTHQTQKSVKSKSIKKSDQDSGSSDENENG

SIGLNASDGSSDGSGAQSSWTKKAVDVDDSPRAVSLWDRVDSTCAQVVHSNPEFPSNQLV

APPAEKETQEHDDKFEDVTMGRDLEISIRRNCDLALEPKDEPLSKTTGIMRQDNSFEKSS

SKWKMKVGKGPLDLSSESPSSKQMHEDGGSSFKAMSSHLQDNREPEAPNTHLKTLDTNEASVKISEELMHVEHSSKRHRGTKDDGTLVRDDRNVLRRSEGSAFSRYNPASNANKISGGNLGSTSLQDNNSQDLIKKTEAAYDCHSNMNESLPHNHRSHVGSNNFDMSSTTENNAFTKPGAPKVSSAGSSSVKHSSFQPLPCDHHNNHASYNLVHVAERKKLPPQCGSSNVYNETIEGNNNTVNYSVNGSVSGSGHGSNGPYGSSNGMNAGGMNMGSDNGAGKNGNGDGSGSGSGSGSGNLADENKISQREAALTKFRQKRKERCFRKKVRYQSRKKLAEQRPRVRGQFVRKTAAATDDNDIKNIEDS

>AT2G46790.1

MGEIVVLSSDDGMETIKNRVKSSEVVQWEKYLPKTVLRVLLVESDYSTRQIITALLRKCC

YKVVAVSDGLAAWEVLKEKSHNIDLILTELDLPSISGFALLALVMEHEACKNIPVIMMSS

QDSIKMVLKCMLRGAADYLIKPMRKNELKNLWQHVWRRLTLRDDPTAHAQSLPASQHNLEDTDETCEDSRYHSDQGSGAQAINYNGHNKLMENGKSVDERDEFKETFDVTMDLIGGIDKRPDSIYKDKSRDECVGPELGLSLKRSCSVSFENQDESKHQKLSLSDASAFSRFEESKSAEKAVVALEESTSGEPKTPTESHEKLRKVTSDQGSATTSSNQENIGSSSVSFRNQVLQSTVTN

QKQDSPIPVESNREKAASKEVEAGSQSTNEGIAGQSSSTEKPKEEESAKQRWSRSQREAA

LMKFRLKRKDRCFDKKVRYQSRKKLAEQRPRVKGQFVRTVNSDASTKS
