# Supplementary material for: A circadian repressor promotes flowering via dual repression: PtTOC suppresses the floral inhibitor PtTFL2 in Pinus tabuliformis
Source: BMC Plant Biol. 2026 Mar 19;26:758. doi: 10.1186/s12870-026-08511-z (PMC13123111; doi:10.1186/s12870-026-08511-z)
Supplement: Supplementary file 1 — Supplementary Material 1. Figure. S1. Expression profiles of 19 RR genes exhibiting generally low expression levels in various organs of P. tabuliformis. The analyzed samples included: hypocotyls (n = 36), seedling needles (n = 90), sapling needles (n = 165), Sapling shoot apex (n = 51), Sapling stem cambium (n = 51), Adult needle(n = 468), Adult shoot apex (n = 69), Adult root (n = 16), Adult stem cambium (n = 51), Adult branch cambium (n = 60), Adult vegetative bud (n = 21), Male cone (n = 60), Female cone (n = 18), Embryo (n = 6), Pollen (n = 3), Ovule (n = 24), Callus (n = 18). [file 12870_2026_8511_MOESM1_ESM.zip › Supplementary/Table S6.docx]

**Table S6 Amino acid sequences of members of the RR gene family in *P. tabuliformis***

**A-ARR**

>Pt3G60260.1

MGRDGGVLLQRVLEAESGSSSSEGSERPVASSQFHVLAVDDSIVDRKVIERLLKISAYKV

TTVDSGRRALEFLGLDEDHSSVDLDNLNVNMIITDYCMPGMTGYDLLKRIKESKKLKEIP

VVIMSSENVVPRINRCLEEGAEEFILKPVKLADVERFKGHIIKGLSKEQQQPASNKRKSM

SEDLDDLPAQPPERRPRLGELSGA*

>PtJG21000.1

MPELTGYDLLKRLKEIKGLKEIPVIIMSSENVPQRIQRCLDEGAEDFIVKPVQRDDIKKI

TGYIKVSKPVATTPICRKRKVSSVVLAGDNSERRPRISGATVA*

>Pt7G06280.1

MPELTGYDLLKRLKEIKGLKEIPVIIMSSENVPQRIQRCLDEGAEDFIVKPVQRDDIKKI

TGYIKVSKPVATTPICRKRKVSSVVLAGDNSERRPRISGATVA*

>Pt0G20440.1

MPELTGYDLLKRLKEIKGLKEIPVIIMSSENVPQRIQRCLDEGAEDFIVKPVQRDDIKKI

TGYIKVSKPVATTPICRKRKVSSVVLAGDNSERRPRISGVTVA*

>Pt6G45400.1

MEEDGVKITFGDSQAITLGEAATDGHEMHVLAVDDCRVDRMVVERLLRSDSVKVTAVDSAAKALELLGVNKEHATATVCTNPLKFSLIITDYCMPELTGYDLLKRLKEIKGLKEIPVIIM

SSENVPQRIQRCLDEGAEDFIVKPVQRDDIKKITGYIKVSKPVATTPICRKRKVSSVVLA

GDNSERRPRISGVTVA*

>Pt6G44810.1

MSVSSAVGRMGGKVCEGVFEEAVAFAGCETHVLAVDDCVIDRKIIERLLETDTVKVTTVD

SPTRALEMLGLSQEPATTPPCKNVFKFSMIITDYCMPEMTGYDLLKKLKETKGLKEIPVV

IISSENVPQIIQRCLDEGAMDFIIKPVQLEDVNKIRRHIIQLQSMHAPLTTVSRKRKALP

TNLAGEASERRPRIKRVRVP*

>PtJG44170.1

MSSTSDHSSQEGESASQLHVLAVDDSVIDRKVIEKLLKNSCYKVTTMDSGRRALEFLGLG

DDQNVANGLQVNMIITDYCMPGMSGHDLLKRVKESSKLKEIPVVVMSSENESSRKRRCLDEGAEEFLLKPVQISDVNRLRDYIRIDHKSDSYCPTGASI*

>PtJG44180.1

MSSTSDHSIEEGESTSQIHVLAVDDSVIDRKVIEKLLKNSCYKVTTMDSGRRALEFLGLG

DNQNVANGLQVNMIITDYCMPGMSGHDLLKRIKESSKLKEIPVVVMSSDNESSRKRRCLE

EGAEEFILKPVQISDVKRLRDYICRDHKSDSYCPAGPSI*

>PtXG20400.1

MVSSRMSSAMRMKKEKNAAFGEHGDELVRCDEMHVLAVDDCLIERKVIEKLLKTNFFKVTSVDSAERALEVLGFHEEQSTCATTNAFKVNMIITDYCMPGMTGYDLLKKVKETKCLKEIPVVIISSENVPQRITRY*

>Pt5G19950.1

MARNESGDAASTSEFHVLAVDDSVLDRKLIEKLLKISSYRVTTVDSVRKALQFLGLEDDE

DDLNVDNIQVNMIITDYCMPGMTGYDLLRRVKESSAVKDIPVVIMSSENVESRISRCMSE

GAEEFLLKPVQLSDVKKLRSHILNKGRSRDQQSTSSSNNKRKTMTDSLSPPAERRPRPSG

LTVA*

>PtJG44210.1

MSSTSDHSVEEGESTSQVHVLAVDDSVIDRKVIEKLLKNSCYKVTTMDSGRRALEFLGLG

DDQNVANVNMIITDYCMPGMSGHDLLKRVKESSKLKEIPVVVMSSDNESSRKRRCLDEGAEEFILKPVQISDVKRLRDYICRDHKSDSYCPTGPSI*

>PtJG44400.1

MERRGWQHSQSRTEDTMAFNSDQSREEVESTAQLHVLAVDDSITDRRVIEKILKNSCYKV

TTVDSVTSALEFLGLGDDHNIIDDSEVNMIITDYSMPGMSGYDLLKRVKESKLKEIPVVV

MSSENLSSRRSR*

>PtJG44410.1

MGRKGSGDSQLRTEDSMPSTSDQSSRQGESTSQLHVLAVDDSTIDRKVIEKLLKNSCYRV

TTVDSGRRALDFLGLGDDHNAVNDLQVNMIITDYCMPGISGYDLLKRVKESSKLKEIPVV

VMSSENVPSRKRRCLDEGAEEFILKPVQISDVKRLREYMSRVQEESSYCPNGSLN*

>PtXG53600.1

MVTRSLLACLGCDVIVVDSGHECLQAMSQARQNFKVLFLDVCMSGMDGYEVAIHIKEMFPNRHERPLLVALTGNTDKETKEKCIKVGMDGVLLKPVSLEKMRSVLVDLLEHGSVCDSI*

>PtXG53610.1

MVTRSLLACLGCDVIVVDSGHECLQAMSQARQNFKVLFLDVCMSGMDGYEVAIHIKEMFPNRHERPLLVALTGNTDKETKEKCIKVGMDGVLLKPVSLEKMRSVLVDLLEHGSVCDSI*

>Pt2G52370.1

MVTRSLLAHLGCDVTVVDSGHECLQAMSQVGHNFKVLFLDVCMSGMDGYEVAIHIQEMFPNRHERPLLVALTGNTDKATKEKCIKVGMDGVLLKPVSLEKMRSVLVDLLEHGSICDSIQRL*

>Pt9G38340.1

MVTRSLLARLGCDVTVVDSGHQCLQAMSQAGQNFKVLFLDVCMPGMDGYEVAIHIQEMFPNRHERPLLVALTGSTDKATKEKCIKVGMDGVLLKPVSLEKMRSVLVDLLEHGSVCDSIQRL*

**B-ARR**

>Pt8G13520.1

MSPLVDRDPQVAKGEAFPVGRSCSDSCCHVGGPTKAAAVTSDEEKFSPSGLQVLVVDDDT

FCLAVLERMLRQCEYNVTVCTRVSQAISLVKENREGFDIVMSDVYLPGEDGFKLLEVVGI

GLGLPVIMMSANGETNVVMRSIRGGACDYLIKPIRTEELRTIWQHVVRRHRQQCHRHDGKDNHGDFSDHGGVLGEFKKRKHSSCQDIKDLNSLKTARVHWTAQLHQRFVKAVNQTGLDTPKRILEIMNVPGLTRENVASHLQKYRLYLKRLSGSIPEPRPVASFQAARDGKFGGTMQIRKRGKPTPSSSSSSSSSSAIKTSPLSLELSRNLDHATLKSLEQFQAYERKLVANRVQMLRGA

ATAAAPSSYMRTNVHVDQGYENGGELRIITHAGRLRKNLQGGGGHRACPADESAAQSFKVDFANLAETKPLTKSFGLSTHQPQFQNPAPTTETPEENDVHKEEAKRLIDETQTIDQEELL

FNNFVEENFPHMKIPSDHDQMPTLIASAGDLSEEYFMLGEAFLPRLGDKEEITQVHIP*

>PtQG11040.1

MRPRVRLAEESRCMKQIENFSPRGMEVLVVDDDPLCLMVLERMLRQCDYNVTTCSRVSEAISMVRERKDRFDLVMSDVYMPNEDGFKLLEFIGLTLDLPVIMMSANGETSMVMKGITHGACDYLVKPVRMEELRNIWQHVVRRRGREYLREESGDSENETSKLVESSESSSKKRKDSQNLDHSSEIIDDISSLKKTRIHWTVQLHQQFVAAVNQLGIDKAVPKRIIEIMGVHGLTRENVA

SHLQKYRLYLKRLSGSIPAPYPVASFQAAEGGKSGGRMHIQPPAKSGASSPVLTKGLNLG

FATSPNSTGSFGIGGIDQGTLKSLEQYRAYEQKRAANRAQVLGGIGIMPSKQSSVGQLPA

ESKGGLQRMASVDLSLLWKAQRDQASSQKPAGLNVGDDLADQSFKKTVKLENVQLEPFPVQPASKHSRAHSMSNVLTDFCNIDPPLTGFQRSGMLKEINELQPMEGISWEGSTSAKGEFKPVEEMSWKGSTSTGGAGAEVGVDTSVDLFDDFVEEYGITSLSPPPTEMDEQSFAGADFEGMGEAGNQDPSPITPDDIADYLFDDFLPRSR*

>Pt9G56450.1

MMMDDKSCVGKLEDECRSIMQIGNFSPIGLKVLVVDDDPLCLLVLERMLRQCNYNVTTCSRVPQAISMVKENRDRFDLVMSEVYLPDEDGFRLLEIVGLGLDLPVIMMSTNGDTSVVMKGITDGAYDYFIKPIRLEELRNIWQHVVHIRGRESLKDDLGECEDCEISDSPDTSSKKRKDT

SSGDFSDEVIDDISSLKRARVHWTVQLHQQFVIAVNQLGIEKAVPKKIVEIMKVQGLSRE

NVASHLQKYRLYLKRLSGAISEPHPVASFQAADDSISGGMMKLKQGEKGVASSSGAKGLN

LGTGVSSSLGVRGLDPSTLKSLQQYRAYQQKLVANRAQIFGGIGVRPPKYSDTAKVPESM

GGSKSGLQRMSSVDMGLLWKAQREQTADENEKQGIANLQNLPQKRSNSSEQRRFRGSHPKHSRTRTMTDLPMIFQSAKPEVTFQKTPPAKSMLQDVDELQLMEDISWLDNTFKPESPRAAVISFDSFVQDFEGSSLSAATIEESNRHAFASTDFEGQRKAGVENEEFFSSGSTDISDYLV

DDLMPHPR*

>Pt1G08590.1

MLHHIFPCIIMSIVMSTNEDTSVVMKGITDGAYDYFIKSIRLEEIRNIWQHVVHIWGRES

LKVDLGECEDCEISDSPDTSSKRRKDTSGGEFFDEVTCDITCIKRARVH*

>Pt3G03540.1

MNSISEMTTMQSLQRSSMSTASSFPSESSGGFDEKSGFNQKSRFPDRFPAGLRVLVVDDD

PICLKIIEKMLRRCLYEVTTCSHATVALSMLRERKGSFDLVISDVYMPDMDGFKLLEHVG

LEMDLPVIMMSADGETSVVMKGIKHGACDYLLKPIRIEELKNIWQHVIRKKRNLGRELEY

SGSAEDSDKNKKGSDDIEYASSVNEGTESNSKTSKRRKETKEEDDDFEQENDDPMSLKKP

RVVWSVELHQQFVNSVNQLGIDKAVPKRILEMMNVHGLTRENVASHLQKYRLYLRRLSEVAQQQSGLNSSFGGTAEVNLGSMCALGRLDLQAFAASGQKSPQTLVAMQAELMGKINAKNGLGISGDTFCLPTAFHGVSSNSLNSLGFGQQLMNNQGGVLQGFPSGLKLKQLSLSQSQPNISSYENGVFSMNDTSPGLPFLPQLSTAAVALRDLDHIYGSNNNLGLDLDDKASMLKLLQQQQLHQTPGPLPHQSGAQLHGQQVLNLPSSSLLGQRTLLNTLSTLSNANSNQGNSLIGPGSAQKNLITPEGHDLTLNACNAYFGIRRMEDVNQSLNSMPVNSVFQPRTVASTPLSSGAEGVMSLPGMNIPRETPEVFNSQALRSSGVNCNALNGFAPGLTQGNVLGCQVPKAAFNQGLGPMVIPTLNPHYGQTSSGLQGLNPGIQDHVLSQSLGFSGKGSNLPNRFTSNDGHLRTISNTPRQSDQSRADTGTRLKDRVSDLMEGTRLAEREFSLDGYN*

>Pt3G51890.1

MLQVLEDCNLWKDFPKGLHVLVVDKDPATLNDIKAKLEACRYRVTTFERSEDAVTALTNP

DSSFHVALIEQAAFGERLDEFDILGAEIRKNIPTISETSKPILPPLPFFSLQFSDKHYTE

NNLGSGSYILYRSIRWLELDTKCYLYLEISLLSYVVMSNTDNTDVMLRAFALGAVEILQK

PLSDDKLKNVWQHAVRKALSTATEPPDDSKNAADEETLWEGEEGGLVIEENPNLKGDIFG

CEKFPAPSTPKRELVDRTYSTLENLDESSVSETTFNFKNEIEETRQMEAAEMILNEDSAS

SGLDEVEKGLDISKIVSLKPNGKRDACTASDLNYPCDQTELDSSFDDFCMSMDGDLLSDI

DPDMFEHFSLDGNEGLDIGPYFLDHCEVQEKDLAEEEALLLAEVSKAETQTQQLMADLPDINNSAFFSDISVKKELSAEDKKTNGRENSKLTSKASQGKRKIKVDWTPDLHRRFVQAVEQLGVDKAVPSRILELMGVNCLTRHNIASHLQKYRSHRKHLLAREAEAATWNHRRQIYATTGTRARPWIAQNGNPLIQPRPSIGFPPMAPASHASFRPLHVWGHPTVDHSSAHMWQNQPMAATAAWPAADGYFWQQPTACTSPWDHNAPASGTPLYPQPLMRLPLAPVPGVPHYMPPVYNGEYYKPENSSAIHPVNIDLPAHLKSSNFHLSKEKVDAAISEVLSNPWTPLPIGLKSPSLESVMAELQRQGISNVPPASA*

>Pt3G71490.1

MVSVAEWKDFPKGLRVLVLDEDSEAAAETKSRLEGFEYVVSTFSNETEALAVLANETNSF

HVALVEVSTGNSCGGFRFLQTACNIPTVMMSNRSCLSTTMKCIALGASEFLQKPISEDKL

KNIWQHVVHKAFSESGTVLSESLKPVKATIVSMLQLGHEHDNVKSKSSQNGDSFVGNKVEAGALHKVQSCMPTRTDSIKQESSIEPCNSEKFPAPSTPQLEQGGRSPCEEDKTLRLDDAT

NLMVGPAHHDTNCGDETIREVRLDAEATSTWSSQVDSPASVKKESQDMYRISLKARISTQ

SGVEPADLTRDQLGNGITSRGTSLHSGQETADVHVVSASGITEEEVGSAEGSKSDDENIE

TEAILSSYVCNEENVDSSTENIKEKKNSAEHGCKKSNISSSKKKFKVEWTMDLHRRFVQA

VEQLGVDQAIPSRILDLMKVDGLTRHNVASHLQKYRSHRRHILPRDDELASRRYWQHFDP

AWTRTKQDESWSRTGNPASGPILAYSPVQLHSTPHGTPVGPPLHVWGHPTIDQSGSHMWQ

QLQVGTPTTWQAQDGSFWKHPGVYADAWGCPTVGMPFYPQPMMKLPSNHGHRSTPKPNSTIQGVSVQYPATLDPLNESLLWEKSINEHPPKEVIDEAFKEALNNPWTPLPLGLKPPSMESVMAELQRQGINKIPPPAS*

>Pt4G04280.1

MATGPAFECSQGLPEFAKKESCSSVNPVNSVSVTSPVMEGVPDQFPAGLRVLVVDDDPTC

LKILEKMLQTCRYEVTTCSRATVALSMLRERRGAFDLVISDVYMPDMDGFKLLEHVGLEMDLPVIMMSADGETSVVMKGIKHGACDYLLKPVRIEALKNIWQHVIRKKRNESKELEHSGSVDDSDRHKKGSDDVEYASSVNEGNNGMWKPSKKRKEAREDEDDGEQDNDDSSNLKKPRVVWSVELHQQFVCAVNQLGIDKAVPKRILELMNVQGLTRENVASHLQKYRLYLRRLSGVGQQQSGFNSSFGGSMEANFGSISSLDRSDLQALAASGQISRQTLAAFQGGLLGRVNGNDVGMSGVDPTFLLQPDLQGLNCSLTDRARFGQPLLNSQRNLLQGLPTGLELKQLSQSHQHMPSFGNLGMPMDDSSPGFPIVQQQLPTASIELGGMGQISSINNNTALNPHNNSLMVQMMQQQQQHPLNHHQQQMPTSAGQQQHPLNHHQQQMPAGSRQQQQSGEQNQNEQLEGVQVLNMPSPNLLQQQMLSNDIGSIANPLSTVGNSSTLLGNTTVGTSVSGQANQIPTGIHGLTLPQSNASVGNGRTPTVDYQNHYHPIMQGNNYSLARAVGTTTPLASVGTCGDMSMAGVGNLGGMSEIVNSHTLRSSNTNFSTLNGFGQNLSQSSKQGWHGQSLAQNSVLSQTGNLVPNSRYSQPFPASQSMNISSTQNKGQVGGFVTCGKGLGLPSRLSTDAGDTRTGTRLQREQSADSALRLKEDGLSSVMPIAKFEGGLLNDHYMQEDLMSVLLKQQQEGVGLAESEFSTDGYQLDVK*

>Pt5G54600.1

MDLSVIMMSANGKTSVVMKGITHGACDYLMKPVRLEEIKNIWQHVVRKRRTESKDHDKLDHGDENDKFNHGPEDGEHGSSANDGTNCSWKLNRKRKEKNDDEDEMQMNMIWKTLQHKNSQELFGQWSCINNL*

>Pt7G57560.1

MLHHIFPCIIMFIVMSTNEDTSVVMKGITDGAYDYFIKSIRLEEIRNIWQHVVHIWGRES

LKVDLGECEDCEISDSPDTSSKRRKDTSGGEFFDEVIGDITCIKRALEG*

>Pt8G42410.1

MGRSEGGLAVVTMQQPPHANTGGVPGNMSMKDGKSNDKVKVMNDEEANDDFPIGMRVLVVDDDPICLLLLESLLRRCKYNVTSCGQAITALNLLRENKDKFDLVISDVYMPDMDGFKLLELVGLEMDLPVIMMSANGETSAVMKGITHGACDYLLKPVRLEELKNIWQHVVRKRRTESKDRDNLDHGDANDKFNHGPEDGEHGSSANDGTDRTWKLNRKRKEQNDDEDDDDDEHDMEDPSTSKKPRVVWSVELHQQFVNAVNSLGIDKAVPKRILELMNVQGLTRENVASHLQKYRLYLKRLSGVASQQGSMGNAFGGGRESSFASLCQVDGIGDLQVLAQSGQLSARALESLQAGVLGRLNGSVGLGLPGLNPSGMLQFASLPGLGSNNSIGRAQGIATVNNPGTAFPCLSTGIELDQLQQKQQITRLGDISSPVDDPAGFRTMQRQLTVTNSLPVGLGGGSSGNIPVNSTNNALVLQLMQQ*

>Pt9G18970.1

MLHHIFPCIIMSVVMSTNEDTSVVMKGITDGAYDYFIKSIRLEEIRNIWQHVVHIWGRES

LKVDLGECEDCEISDSPDTSSKRRKDTSGGEFFDEVTGDITCIKRA*

>Pt9G28610.1

MLHHIFPCIIMSIVMSTNEDTSVVMKGITNGSYDYFIKSIRLDKIRNIWQHVVHIWGIES

LKVDLGECGDCEISDSPDTSSKRRKDTSGGEFFDEVAVKL*

>Pt9G55570.1

MMMDDKSCVGKLEDECRSIMQIGNFSPIGLKVLVVDDDPLCLLVLERMLRQCNYNVTTCSRVPQAISMVKENRDRFDLVMSEVYLPDEDGFRLLEIVGLGLDLPVIMMSTNGDTSVVMKGITDGAYDYFIKPIRLEEIRNIWQHVVHIWGRESLKVDLGECEDCEISDSPDTSSKKRKDT

SGGDFFDEVIDDISSLKRARVHWTMQLLQQFVSTVDQLGIDRAIPKKIIKIMKFEGLSLE

NVASHLQKYRLHLKRLSGAISEPHPVASFQAADDSISGGMMKLKQGEKGVASSSGAKGLN

LGTGVSSSLGVRGLDPSTLKSLQQYRAYQQKLVANRAQIFGGIGVRPPKYSDTAKVPESM

GGSKSGLQRMSSVDMGLLWKAQREQTADENEKQGIANLQNLPQKRSNSSEQRRFRGSHPKHSRTRTMTDLPMIFQSAKPEVTFQKTPPAKSMLQDVDELQPMEDISWLDNTFKPESPRAAVISFDSFVQDFEGSSLSAATIEESNRHAFASTDFEGQRKAAVENEEFFSSGSTDISDYLV

DDLMPHPR*

>PtXG23320.1

MLHHIFPCIIMSIVMSTNEDTSVVMKGITDGAYDYFIKSIRLEEIRNIWKHVVHIWGRES

LKVDLGECEDCEVSDSPDTSSKRRKDTSGGEFFDEVTCDITCIKRARVH*

>PtXG42450.1

MLRENKGAFDLVISDVYMPDMDGFKLLEQVGLEMDLPVIMMSADGGTSTVMKGIKHGACDYWIKPIRLEELKNIWQCVIRKKRNEPKDFDLSGSFEDNDRHRKGSDDVDYASSVNEGTDGNWKLLKKRKEAKEEEDDGEYDNDDPFASKKPRVV*

**Pseudo-RR**

>Pt3G36450.1

MSKASGAMSTWRYKRDADPKQSEDGLAKRVKFVVNLESGGENDGEQTLGRVAEKVSFRYVDGSNVQKGSGTMHNKEVVRPKNMQVRPDAEGNNRLAEMNRHGRQDQKEARGGIMGDGQELSEQDESRDDDGMIDVGDGDWHLVDANLLSRRLPHQQPPQPQGSVIRWERFLPQRCLKVLLVENDDSTRQVVSALLRNCSYEVVAVANGQQAWKLLEDPSNHFDIVLTEVVMPCLSGIGLLCKIMSRSTCKNIPVIMMSSHDSMGTVFKCLSKGAVDFLVKPVRKNELKNLWQHVWRRCQSSSDSGGETGSQSQKVTRPNGTADPDNNTGSNDESDDASIGLNNRDGSDNGSGTQVSWTKRVVEVESPQHRTEWGLSQPHSSAVGQVIQQKVETAQNGWMQMAVTSGECQDKKPLDYAMGQDLEIAAPRQTDAELEHQEEEKALYAQTFGREENAPLANSVEGVENSEGPVESGDSPSSEVAANKAADLIGVIANKPNSGNSKLEDNKSGEDLDNDKTGTDFKHLFLELTLKRPRQNGKEDGEPEDRHVLRQSGVSAFSRYNTGGCQFSNPSGDTVPPNSLAKGYMPSGSLESEKTGPLHAAVGVERVGSSKGSGGPDQSTPALMHNQLSQSSNNQDLGSSVVGPSGQDMFPVPKPINDETVSAPNTCRQVGKPMSIHPCRAMPYDTVAAPRVYGSAIHPLYYSHTGASSLWGPEAQHITDRGDVCNNSNNGQIEQQLLLPRNHQTQVHHHHHHHVQHVHYHEHHHQTHQHKHNHTHTHDHPKQDEQTKNNLSITPPLCGSSNMSGNNAPDGNNGQSGSNNGYGSSGNGNGSANGSASGSNNGSNGQNGYSNGQNSAAVTPPGTNGESDTGIGAVKSSTGGASGTGSGSGVDINRSAQREAALTKFRQKRKERCFEKKVRYQSRKKLAEQRPRVRGQFVRQTAHETANGEVD*

>Pt5G04390.1

MGKGSISNSDAGSSVLDRSCVRILLCDKDPTNSQQLLELLRKCSYQVTAVSTAREVVSVL

NTEGREIDLILAEVDLPKSKGFKMLKYITRSTCLQRIPIVMMSAQDEVAVVMKCLKLGAA

DYLVKPLRINELLNLWMHMWRRRRMLGLADKNIISKNLGHDLDMLVSDLSDSNTNSTNLFSDDTNDKKVRSHAGPEISTQVTPPECEISLCLPLGIMSLALVQLQSHDSPKLELSLKRSS

EGPPEEPELGSLAGKFLSYPKRSEVKFGGASAFLTYVNASVQANRTPNQISVGENKASQQ

ETAIPEKHGVMGPPSTDNRPIGLSHSSEAVKSSNRTEVIPCEEHRRDRLETRSCNVSMSP

EIPIGQVAPAGEQFPMVQGGLPNEGSGMNNHDISSLPASHFLPGMMNHSMSASMHLCHGV

HHDVGSHGAPRLIPFHTFQPCHGMPVNATMPYYPYGFVVAPATIGSSHAWPGMANLSVSE

PKITQVERREAALNKFRQKRKDRCFDKKIRYVSRKRLAEQRPRIRGQFVRQTNDMEAAGANGVVYGVDSSEDEDDGYVHGSGELRLTSSPESLAGDTENAI*
