# Supplementary figures and images for: A circadian repressor promotes flowering via dual repression: PtTOC suppresses the floral inhibitor PtTFL2 in Pinus tabuliformis
Source: BMC Plant Biol. 2026 Mar 19;26:758. doi: 10.1186/s12870-026-08511-z (PMC13123111; doi:10.1186/s12870-026-08511-z)

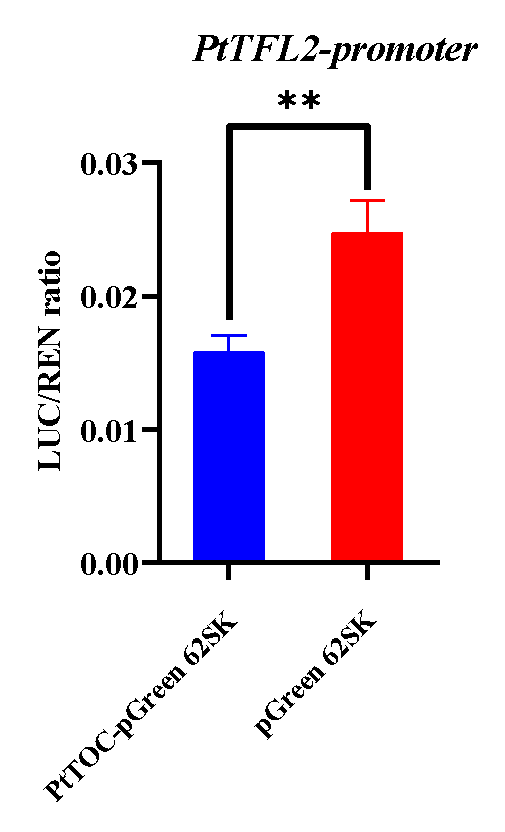

Supplement: Supplementary file 1 — Supplementary Material 1. Figure. S1. Expression profiles of 19 RR genes exhibiting generally low expression levels in various organs of P. tabuliformis. The analyzed samples included: hypocotyls (n = 36), seedling needles (n = 90), sapling needles (n = 165), Sapling shoot apex (n = 51), Sapling stem cambium (n = 51), Adult needle(n = 468), Adult shoot apex (n = 69), Adult root (n = 16), Adult stem cambium (n = 51), Adult branch cambium (n = 60), Adult vegetative bud (n = 21), Male cone (n = 60), Female cone (n = 18), Embryo (n = 6), Pollen (n = 3), Ovule (n = 24), Callus (n = 18). [file 12870_2026_8511_MOESM1_ESM.zip › Supplementary/Fig. S2.tiff]

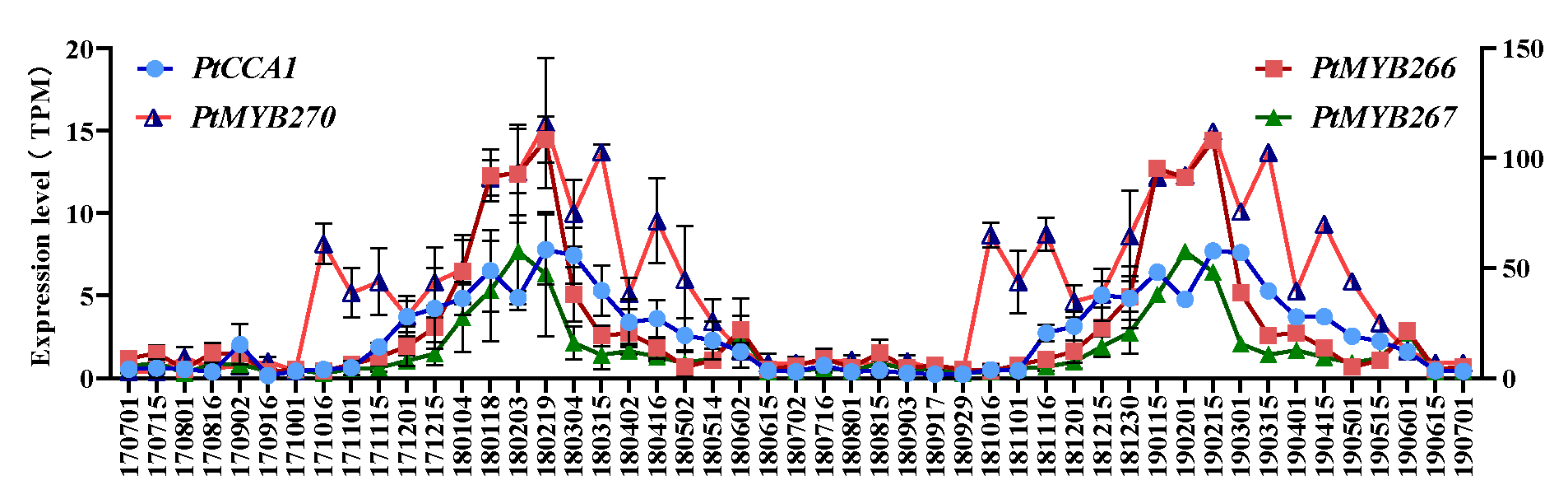

Supplement: Supplementary file 1 — Supplementary Material 1. Figure. S1. Expression profiles of 19 RR genes exhibiting generally low expression levels in various organs of P. tabuliformis. The analyzed samples included: hypocotyls (n = 36), seedling needles (n = 90), sapling needles (n = 165), Sapling shoot apex (n = 51), Sapling stem cambium (n = 51), Adult needle(n = 468), Adult shoot apex (n = 69), Adult root (n = 16), Adult stem cambium (n = 51), Adult branch cambium (n = 60), Adult vegetative bud (n = 21), Male cone (n = 60), Female cone (n = 18), Embryo (n = 6), Pollen (n = 3), Ovule (n = 24), Callus (n = 18). [file 12870_2026_8511_MOESM1_ESM.zip › Supplementary/Fig. S3.tiff]

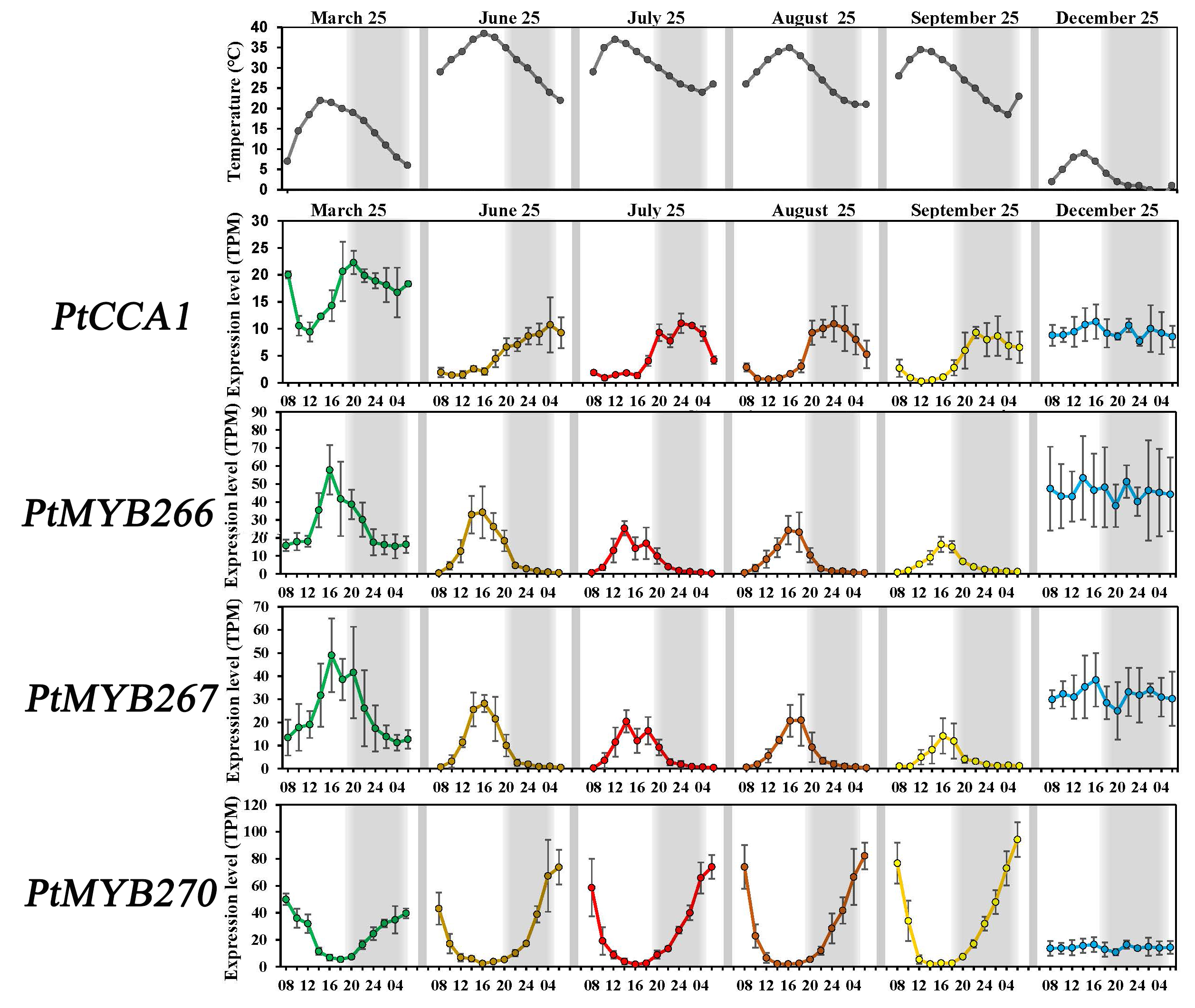

Supplement: Supplementary file 1 — Supplementary Material 1. Figure. S1. Expression profiles of 19 RR genes exhibiting generally low expression levels in various organs of P. tabuliformis. The analyzed samples included: hypocotyls (n = 36), seedling needles (n = 90), sapling needles (n = 165), Sapling shoot apex (n = 51), Sapling stem cambium (n = 51), Adult needle(n = 468), Adult shoot apex (n = 69), Adult root (n = 16), Adult stem cambium (n = 51), Adult branch cambium (n = 60), Adult vegetative bud (n = 21), Male cone (n = 60), Female cone (n = 18), Embryo (n = 6), Pollen (n = 3), Ovule (n = 24), Callus (n = 18). [file 12870_2026_8511_MOESM1_ESM.zip › Supplementary/Fig. S4.jpg]
